# Frenkel Excitons in Vacancy-Ordered Titanium Halide Perovskites (Cs_2_TiX_6_)

**DOI:** 10.1021/acs.jpclett.2c02436

**Published:** 2022-11-22

**Authors:** Seán R. Kavanagh, Christopher N. Savory, Shanti M. Liga, Gerasimos Konstantatos, Aron Walsh, David O. Scanlon

**Affiliations:** † Thomas Young Centre and Department of Chemistry, University College London, 20 Gordon Street, LondonWC1H 0AJ, U.K.; ‡ Thomas Young Centre and Department of Materials, Imperial College London, Exhibition Road, LondonSW7 2AZ, U.K.; § ICFO, Institut de Ciencies Fotoniques, The Barcelona Institute of Science and Technology, Castelldefels, 08860Barcelona, Spain; ∥ ICREA, Institució Catalana de Recerca i Estudis Avançats, 08010Barcelona, Spain

## Abstract

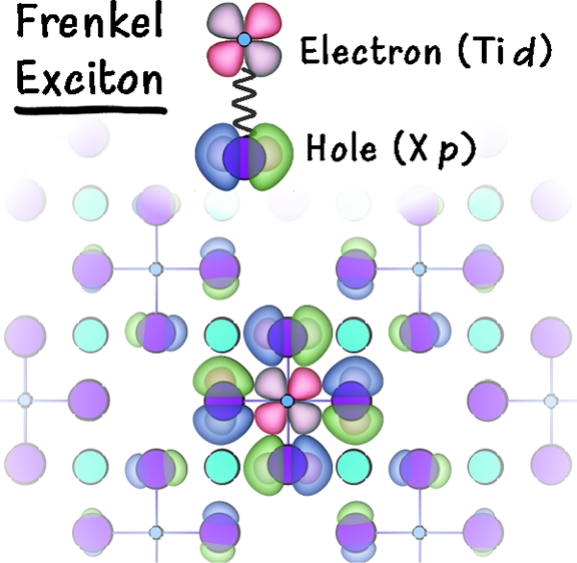

Low-cost, nontoxic, and earth-abundant photovoltaic materials are long-sought targets in the solar cell research community. Perovskite-inspired materials have emerged as promising candidates for this goal, with researchers employing materials design strategies including structural, dimensional, and compositional transformations to avoid the use of rare and toxic elemental constituents, while attempting to maintain high optoelectronic performance. These strategies have recently been invoked to propose Ti-based vacancy-ordered halide perovskites (A_2_TiX_6_; A = CH_3_NH_3_, Cs, Rb, or K; X = I, Br, or Cl) for photovoltaic operation, following the initial promise of Cs_2_SnX_6_ compounds. Theoretical investigations of these materials, however, consistently overestimate their band gaps, a fundamental property for photovoltaic applications. Here, we reveal strong excitonic effects as the origin of this discrepancy between theory and experiment, a consequence of both low structural dimensionality and band localization. These findings have vital implications for the optoelectronic application of these compounds while also highlighting the importance of frontier-orbital character for chemical substitution in materials design strategies.

Perovskite-inspired materials aim to replicate the exceptional optoelectronic performance of lead halide perovskites (LHPs), while avoiding issues of toxicity and operational stability.^1^ For decades, the standard materials design approach for identifying novel inorganic semiconductors has been chemical substitution, in which the undesirable elemental constituents (e.g., toxic Pb^2+^ in LHPs) are replaced by more favorable counterparts, while retaining the same structural motifs. For example, in the diamond-cubic crystal family, research moved from group IV elements Si and Ge to II–VI compounds like CdTe, to yield direct rather than indirect electronic band gaps, and then further splitting into the I–III–VI_2_ (e.g., CuInSe_2_) and I_2_–II–IV–VI_4_ families (e.g., Cu_2_ZnSnS_4_), to give earth-abundant compositions. While strategies such as dimensional modification^2^ and disorder engineering^3,4^ have recently gained in popularity, elemental substitution remains the prevailing design approach.

Strategies for replacing the divalent B-site cation in halide perovskites, while retaining the BX_6_ octahedral motif, have led to the exploration of A_2_BB′X_6_ double perovskites with a pair of monovalent and trivalent cations at the B and B′ sites,^1,5,6^ as well as the A_3_B_2_X_9_ “vacancy-ordered perovskites”, in which a trivalent B cation is combined with a 1/3 vacancy of the B site to satisfy electroneutrality.^7−9^ Issues of indirect and/or large band gaps in these materials have led to the emergence of A_2_BX_6_ vacancy-ordered double perovskites (VODPs), in which now the combination of a tetravalent cation and a 50% vacancy of the B site is employed, giving a checkerboard arrangement (Figure 1).^10−12^ Also known as defective or tetravalent perovskites, these compounds are actually some of the decomposition products of conventional ABX_3_ perovskites, for example, CsSnI_3_, which breaks down to form Cs_2_SnI_6_.^13^

**Figure 1:**
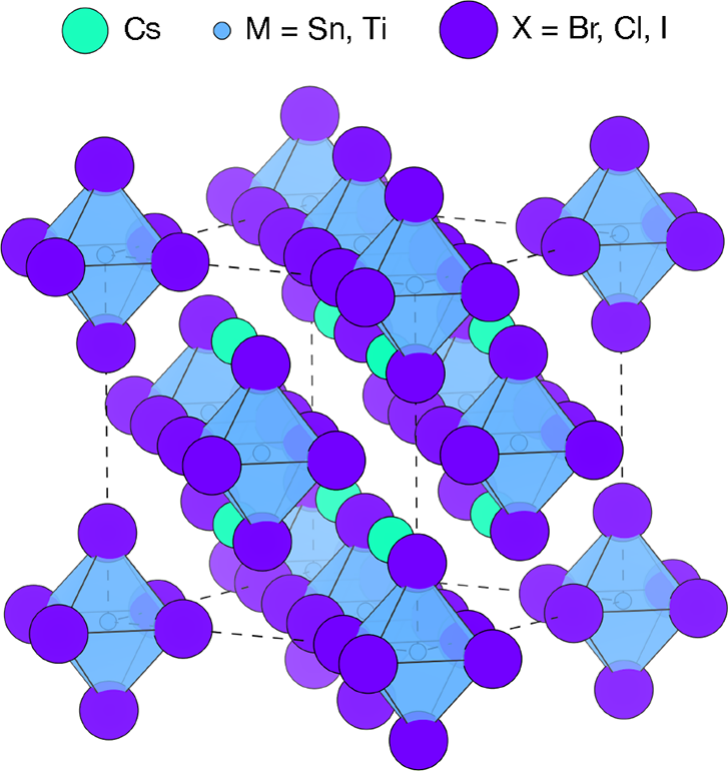
Crystal structure of Cs_2_BX_6_ vacancy-ordered perovskites, in the conventional cubic unit cell (space group *Fm*3̅*m*). Cs atoms are colored green, M cations blue, and halide anions (X) purple.

A_2_BX_6_ vacancy-ordered perovskites have shown promise for optoelectronic applications, with good stability under air, moisture, light, and thermal stresses, as well as being solution-processable and nontoxic.^11,14,15^ As with the single and double perovskites, the frontier orbitals of the B cation and X anion govern the conduction and valence band-edge properties, respectively. This combination allows tunability in the energy gap, absorption profile, and carrier effective masses, for example. The A-site species, on the contrary, is a large monovalent cation such as Cs^+^, which behaves as a spectator, dictating the spacing between BX_6_ octahedra but with no direct contribution to the band edges. A crucial difference from the ABX_3_ perovskite template is the lack of corner-sharing BX_6_ octahedra caused by vacancy introduction. Consequently, the crystal structure is comprised of isolated octahedra and thus an effective zero-dimensional (0D) framework, with this dramatic reduction in connectivity being a key factor in the properties of this material family.^10,11,14^ Research efforts in this area initially focused on the Sn-based compounds (A_2_SnX_6_)^16^ but have since expanded so that a range of tetravalent species have successfully been implemented in A_2_BX_6_ materials, including Te, Pd, Zr, and Pt.^17−20^ While some of these compounds have shown promise as potential white light and tunable emitters, the Sn- and Ti-based materials have shown the most promising results in the context of solar photovoltaic applications and thus received a majority of the research attention. Cs_2_SnI_6_ was originally used as a hole-transporting layer in dye-sensitized solar cells, for instance, achieving efficiencies of 8%,^16^ while a Cs_2_TiBr_6_ photovoltaic device demonstrated a modest efficiency of 3%.^14^ The poor performance of these materials has been attributed to relatively weak visible light absorption and indirect band gaps.^21^

As issues of defect intolerance and operational instability are becoming apparent for Cs_2_SnI_6_,^22^ there is growing interest in Ti-based compounds. The effects on structure, stability, and electronic properties in going from the group 14 *d*^10^*s*^0^ Sn^4+^ to group 4 *d*^0^*s*^0^ Ti^4+^ cations have been probed;^10,11,23^ however, the performance limits of these materials remain an open question. Notably, while theoretical methods are found to successfully reproduce the experimental electronic structure of the Te- and Sn-based compounds,^11^ a major discrepancy exists for the *d*^0^ Ti-based compounds,^10,14,21,23−29^ with severe overestimation of the experimental band gap by both hybrid density functional theory (DFT) and Green’s function (*GW*) methods. So extreme is the error that these theoretical methods actually yield qualitatively incorrect relative band gap energies for the Sn versus Ti compounds, as we show in this study.

Through in-depth computations including explicit electron–hole interactions via the Bethe–Salpeter equation (BSE), we resolve the Ti perovskite discrepancy and reveal strong excitonic effects as the origin. Electron–hole interactions result in significant renormalization of the lowest-energy electronic excitation, as well as qualitative reshaping of the optical absorption spectrum, finally reconciling computational predictions with experimental measurements. We elucidate the origins of this behavior and highlight the implications of strong exciton binding for applications of these materials in optoelectronic devices.

The crystal structure of the Cs_2_BX_6_ (B = Sn or Ti; X = Cl, Br, or I) family of vacancy-ordered perovskites is shown in Figure 1. The low structural dimensionality of this family is expected to produce behavior similar to that of the corresponding [BX_6_]^2–^ molecular salts.^16,24^ One consequence of this “molecular” crystal structure is the possibility for intermolecular interactions, such as London dispersion, between the localized octahedra. Table 1 corroborates this hypothesis, showing contraction of the calculated lattice parameters upon inclusion of dispersion corrections in the model, demonstrating the presence of important van der Waals (vdW) bonding contributions. Indeed, geometry optimization with hybrid DFT excluding dispersion corrections consistently overestimates the experimental lattice parameters by ∼3%, whereas inclusion of vdW effects gives lattice constants with errors of <1% in all cases. Semilocal DFT including dispersion corrections (PBE+D3) was also found to accurately reproduce the experimental lattice constants (Table S1). The change in the lattice parameter (*Δa*_D3_) is consistent within each halide subclass, irrespective of the B-site identity (Sn or Ti), reflecting the expected interoctahedral (BX_6_–BX_6_) rather than intra-octahedral (B–X) origin of these vdW interactions. Moreover, we demonstrate the importance of dispersion interactions between the BX_6_ molecular blocks on the electronic properties, showing the calculated energy band gap to shift by 0.04–0.31 eV in the optimized crystal structure. There is an increasing sensitivity of the band gap to the lattice parameter as we move down the halogen group (Cl → Br → I), as the through-space B–X and X–X interactions in the conduction and valence bands strengthen with larger X *p* orbitals, also explaining the reduced band gap shifts for B = Ti due to the more localized d orbitals. We further note a sensitivity of the electronic band gap on the DFT functional choice for geometry optimization, with a 0.4 eV lower (−40%) band gap obtained for Cs_2_SnI_6_ using semilocal DFT (PBEsol) for structure relaxation.^30^ Hybrid DFT including dispersion corrections was employed for all further DFT calculations in this study.

**Table 1:** Calculated Cubic Lattice Parameters and Electronic Band Gap Shifts (*ΔE*_g, D3_) for Cs_2_BX_6_ (B = Sn or Ti; X = Cl, Br, or I) Using Hybrid DFT Including Spin–Orbit Coupling (HSE06+SOC), with and without Explicit Inclusion of vdW Dispersion Interactions (D3 correction)^a^

	Cs_2_SnCl_6_	Cs_2_SnBr_6_	Cs_2_SnI_6_	Cs_2_TiCl_6_	Cs_2_TiBr_6_	Cs_2_TiI_6_
*a*_HSE06_ (Å)	10.65	11.15	11.95	10.51	10.99	11.76
*Δa*_HSE06_ (%)	2.8	3.5	2.7	2.6	2.9	2.3
*a*_HSE06+D3_ (Å)	10.32	10.78	11.54	10.18	10.62	11.32
*Δa*_HSE06+D3_ (%)	–0.4	0.1	–0.9	–0.6	–0.6	–1.5
*a*_Exp_ (Å)	10.36	10.77	11.64	10.24	10.68	11.5
*Δa*_D3_ (Å)	–0.33	–0.37	–0.41	–0.33	–0.37	–0.44
*ΔE*_g, D3_ (eV)	–0.14	–0.23	–0.31	–0.04	–0.08	–0.15

a Lattice parameter errors (*Δa*) given with respect to experimental values. Experimental values taken from refs (31) and (32) for Cs_2_SnCl_6_, refs (32) and (33) for Cs_2_SnBr_6_, refs (30), (32), and (34−36) for Cs_2_SnI_6_, refs (23) and (37) for Cs_2_TiCl_6_, refs (21), (23), and (37) for Cs_2_TiBr_6_, and ref (38) for Cs_2_TiI_6_, matching with our measured values (Section S1.6)

The electronic band structures, densities of states, and charge densities at the valence band maximum (VBM) and conduction band minimum (CBM) for Cs_2_TiI_6_ and Cs_2_SnI_6_ are shown in Figures 2 and 3. While Cs_2_SnX_6_ compounds exhibit direct electronic band gaps at Γ, Cs_2_TiX_6_ compounds have indirect gaps with the CBM at the X high-symmetry *k*-point and the VBM remaining at Γ, in agreement with experimental studies.^15,21^ The direct/indirect gap energy difference (Δ) is relatively small, however, with Δ values of 0.06, 0.07, and 0.04 eV for the I, Br, and Cl isomorphs, respectively, calculated using HSE06+SOC. As previously noted,^10,11^ the VBM and CBM electronic levels follow that predicted by BX_6_^2–^ crystal field splitting molecular-orbital diagrams, with a *t*_2g_^*^(π) Ti *d*–X *p* CBM for Cs_2_TiX_6_ (*d*_*xy*_, *d*_*xz*_, *d*_*yz*_; 3-fold degenerate at Γ) and an *e*_g_^*^(σ) band just above (d_*z*^2^_ and d_*x*^2^–*y*^2^_), a single *a*_1g_^*^(σ) Sn *s*–X *p* CBM for Cs_2_SnX_6_, and nonbonding X *pt*_2*g*_(π) states at the VBM in all cases (Figures 2 and 3c). The centrosymmetric crystal structure and equal (gerade) parity with respect to inversion for the VBM and CBM states (Figures 2 and 3b,c) result in a dipole-forbidden transition at the direct band gap. Consequently, the symmetry-allowed direct band gap (*E*_g, allowed_) corresponds to the vertical transition from the second-highest valence band at Γ [*t*_1*u*_ (Γ_15_) symmetry; ψ_VBM–1_] to the CBM.

**Figure 2:**
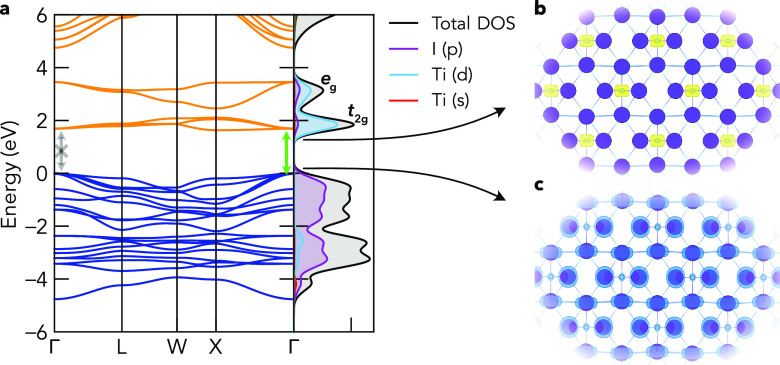
(a) Electronic band structure of Cs_2_TiI_6_ calculated with hybrid DFT including spin–orbit coupling (HSE06+SOC), alongside a vertical plot of the orbital-projected electronic density of states. Faded gray and green arrows indicate the lowest-energy symmetry-forbidden and allowed electronic transitions, respectively (*ΔE*_*t*_1*g*_/*t*_1*u*__ = 0.02 eV). Valence band in blue, conduction band in orange, and valence band maximum (VBM) set to 0 eV. Ti *d* conduction bands are labeled with their crystal field orbital symmetries. Charge densities at the (b) conduction band minimum (CBM) and (c) VBM. Unoccupied states in yellow and occupied states in blue.

**Figure 3:**
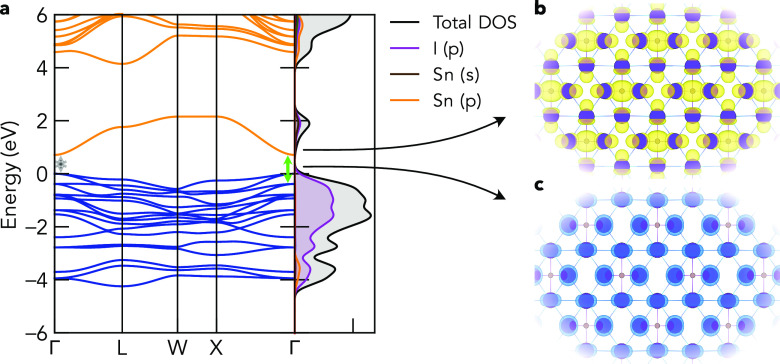
(a) Electronic band structure of Cs_2_SnI_6_ calculated with hybrid DFT including spin–orbit coupling (HSE06+SOC), alongside a vertical plot of the orbital-projected electronic density of states. Faded gray and green arrows indicate the lowest-energy symmetry-forbidden and allowed electronic transitions, respectively (*ΔE*_*t*_1*g*_/*t*_1*u*__ = 0.38 eV). Valence band in blue, conduction band in orange, and VBM set to 0 eV. Charge densities at the (b) conduction band minimum (CBM) and (c) valence band maximum (VBM), using the same isosurface levels that were used for Cs_2_TiI_6_. Unoccupied states in yellow and occupied states in blue.

The halide p valence band is similar for both compounds, though with a slightly wider bandwidth (∼0.5 eV) for the Ti analogues (Figures 2 and 3 and Figures S3–S8) due to a significantly reduced anion–anion distance (*d*_I–I_ = 4.03 Å for Cs_2_SnI_6_ vs *d*_I–I_ = 3.87 Å for Cs_2_TiI_6_) and wider interaction range between the cation valence orbitals (Ti *s* and *d*) with anion *p* states in the lower valence band, compared to those of Sn *p*. This is a consequence of reduced M–X bond lengths (2.73 Å vs 2.85 Å) and lattice parameters for B = Ti versus Sn (Table 1), aided by the reduced ionic radius of Ti^4+^ versus that of Sn^4+^, resulting in a much lower energy difference between the *t*_1*g*_ ψ_VBM_ and *t*_1*u*_ ψ_VBM–1_ for B = Ti versus Sn, with Δ*E*_*t*_1*g*_/*t*_1*u*__ = 0.02 eV/0.38 eV, 0.07 eV/0.30 eV, and 0.07 eV/0.07 eV for B = Ti/Sn and X = I, Br, Cl (using HSE06+SOC). Another consequence is that, in contrast to the electron masses, the hole effective masses are actually larger for Cs_2_SnX_6_ than for Cs_2_TiX_6_ (Table 2). Unlike conventional perovskites and many other “perovskite-inspired” materials that retain the partially oxidized, filled valence subshell of the B cation (yielding antibonding character at the VBM^1,39,40^), the fully oxidized B^4+^ in A_2_BX_6_ means we have a less dispersive, nonbonding VBM,^4^ yielding heavier hole masses (particularly for X = Br or Cl) and aiding carrier localization. In contrast, the conduction band of the Sn analogues is relatively disperse with low electron effective masses (Table 2) due to strong mixing and delocalization of the Sn *s* and X *p* states, while extremely flat bands are found for B = Ti due to weak Ti *d*–X *p* mixing and localized, isolated Ti *d* states. The band structures of the bromide and chloride isomorphs are included in Figures S3–S8, showing similar results, though with larger band gaps and reduced dispersion as X changes from I to Br to Cl. Further analysis of the electronic structure is provided in Section S2.

**Table 2:** Calculated Direct (*E*_g, direct_) and Direct-Allowed Band Gaps (*E*_g, allowed_), Effective Masses (),^a^ High-Frequency Dielectric Constants (ε_∞_), and Wannier–Mott Model Exciton Binding Energies (*E*_ex, Wannier_) for Cs_2_BX_6_ (B = Sn or Ti; X = Cl, Br, or I), Using Hybrid DFT Including Spin–Orbit Coupling (HSE06+SOC) and Comparison to Experimentally Reported Band Gap Ranges^b^

	Cs_2_SnCl_6_	Cs_2_SnBr_6_	Cs_2_SnI_6_	Cs_2_TiCl_6_	Cs_2_TiBr_6_	Cs_2_TiI_6_
*E*_g, direct_ (eV)	4.10	2.39	0.71	3.68	2.75	1.69
*E*_g, allowed_ (eV)	4.38	2.70	1.09	3.79	2.84	1.71
*E*_g, exp_ (eV)	4.4–4.9	2.7–3.3	1.25–1.3	2.8–3.4	1.8–2.3	1.0–1.2
(*m*_0_)	0.55	0.38	0.26	3.5	2.7	1.8
(*m*_0_)	2.2	1.3	0.78	2.2	0.90	0.55
ε_∞_	2.86	3.37	4.54	3.26	3.84	5.08
*E*_ex, Wannier_ (eV)	0.73	0.35	0.13	1.73	0.62	0.22

a values are computed from the harmonic mean over directions and light/heavy bands for the effective masses. Values of >1 are given to one decimal place.

b Experimental band gap values taken from refs (31), (32), (42), and (43) for Cs_2_SnCl_6_, refs (32), (35), (36), and (42−44) for Cs_2_SnBr_6_, refs (16), (17), (31), (32), (35), (42), and (45) for Cs_2_SnI_6_, refs (23) and (37) for Cs_2_TiCl_6_, refs (14), (15), (21), (23), (24), (37), (38), and (46) for Cs_2_TiBr_6_, and refs (24) and (37) for Cs_2_TiI_6_.

The electronic properties of the Cs_2_BX_6_ family are listed in Table 2. To illustrate the expected trends in exciton binding based on band structure and dielectric screening, the Wannier–Mott model binding energies are also included, calculated using the average carrier effective masses () and high-frequency dielectric constants () from hybrid DFT (HSE06+SOC) according to^41^
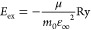
1where  is the reduced mass of the electron–hole pair, Ry is the Rydberg energy (13.6 eV), and *m*_0_ is the electron rest mass.

From Table 2, we witness the typical trend of a larger band gap with smaller and more electronegative halogen anions [*E*_g_(Cl) > *E*_g_(Br) > *E*_g_(I)], as observed across the perovskite (-inspired) family.^1,10^ Typically, the smaller the B-site atom, the smaller the band gap in the A_2_BX_6_ family.^10,47^ This is the case experimentally here, with all Ti isomorphs having experimentally measured band gaps that are smaller than those of their Sn counterparts. The opposite trend is found in the computed band gaps, for which hybrid DFT incorrectly predicts larger gaps for the Ti compounds (except for X = Cl). While the direct-allowed gaps computed by hybrid DFT mostly coincide with the lower end of experimental ranges for Cs_2_SnX_6_, neglecting Wannier–Mott-predicted exciton binding, the entirely opposite trend is found for each Cs_2_TiX_6_ isomorph, with a consistent severe overestimation of the experimental band gap. Notably, screened hybrid DFT (HSE06) tends to slightly underestimate rather than overestimate semiconductor band gaps, with this underestimation typically worsening with larger band gaps.^48,49^ The error in predicted band gaps for Cs_2_TiX_6_ results in qualitatively incorrect relative band gap energies for Cs_2_SnX_6_ versus Cs_2_TiX_6_ (X = I or Br).

A dielectric-dependent hybrid functional approach was also tested, which can improve the description of dielectric screening from hybrid DFT with fixed exchange (e.g., HSE06) and give reduced band gap prediction errors,^49−51^ though this only slightly reduced the hybrid DFT gap for Cs_2_TiI_6_ by 0.04 eV (α_SCF_ = 24%), still giving a significantly overestimated band gap with a relative error Δ*E*_g_ of ∼70%. Even using the computationally intensive *GW* approximation, typically a gold standard for predicting band gaps,^52,53^ the calculated quasiparticle gaps in fact show far worse overestimation [for both Cs_2_TiX_6_, as previously noted by Cucco et al.,^10^ and Cs_2_SnX_6_ (Section S3)]. These observations suggest the presence of physical interactions in Cs_2_TiX_6_ that are not captured in these single-particle electronic models.

This major experiment–-theory discrepancy is witnessed in reported values across the literature^10,14,21,23−29^ but has not been addressed until now. In many cases, semilocal DFT (known to severely underestimate semiconductor band gaps)^48^ has been employed to yield fortuitous error cancellation and thus theoretical values closer to experiment. As we show in this work, however, semilocal DFT predicts qualitatively incorrect relative band gaps [even finding Cs_2_SnI_6_ to be metallic, for example (Table S3)], alongside incorrect absorption spectra, thus being unsuitable for modeling the electronic structure of A_2_BX_6_ compounds.

Using the Wannier–Mott effective mass model (Table 2),^54^ we find large exciton binding energies, particularly for the Ti compounds (due to flat bands and heavy carrier masses), suggesting strong electron–hole interactions in these materials. Moreover, the large Wannier–Mott binding energies, isolated octahedra, low dielectric screening, and highly localized d orbitals suggest that strongly bound excitons may be formed for Cs_2_TiX_6_.^41^ To explore this hypothesis, we extend our model using *GW* to calculate quasiparticle eigenvalues and include electron–hole interactions via the Bethe–Salpeter equation (BSE). The optical absorption spectrum calculated with this approach, alongside that obtained from hybrid DFT and the experimental data, is shown in Figure 4.

**Figure 4:**
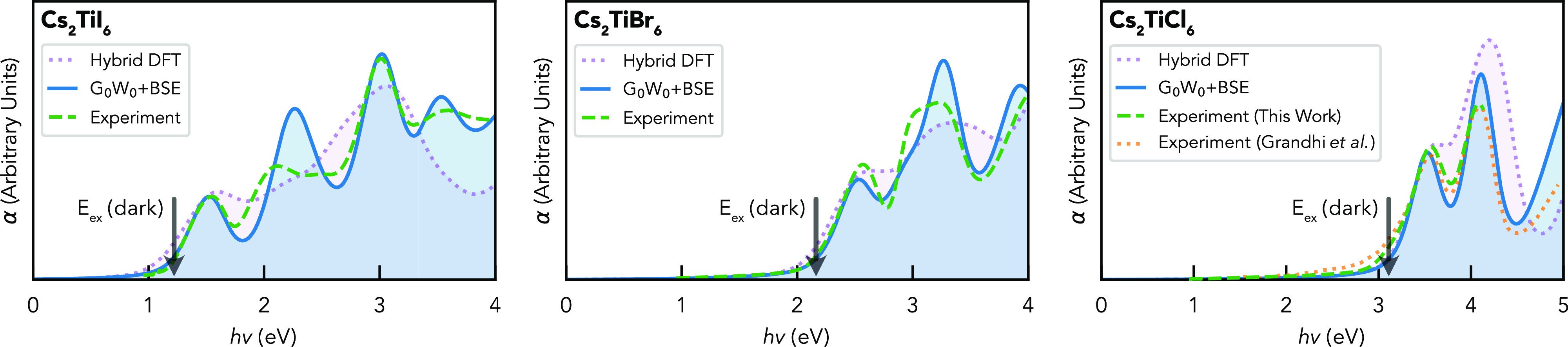
Optical absorption spectra of Cs_2_TiI_6_, Cs_2_TiBr_6_, and Cs_2_TiCl_6_ (from left to right, respectively), calculated with both hybrid DFT (dotted violet) and the *G*_0_*W*_0_ + BSE method (solid blue), alongside the experimental data from ultraviolet–visible spectroscopy (dashed green). To directly compare the spectral shapes, calculated curves have been rigidly shifted to match the experimental absorption onset (unshifted results shown in Section S3).

Electron–hole interactions are found to dramatically red-shift (Tables S2 and S3 and Figure S10) and qualitatively alter the absorption spectra for Cs_2_TiX_6_, now yielding excellent agreement with the peaked experimental spectra. Analysis of the electronic states reveals the lowest-energy bright exciton peak to originate from the *t*_1*u*_(π + σ) → *t*_2*g*_(*d*) electronic transition as expected, i.e., from the second-highest valence band (ψ_VBM–1_) at Γ (Figure 2) to the CBM. The lowest-energy dark excitonic state, indicated by the arrows in Figure 4 and located 0.3–0.4 eV below the first bright peak, corresponds to the symmetry-forbidden *t*_1*g*_(π) ψ_VBM_ → *t*_2*g*_(*d*) ψ_CBM_ transition mentioned previously. While improved agreement with the experimental spectra is found for all Cs_2_TiX_6_ isomorphs, smaller changes in the spectral shapes are noticed when X = Br and Cl. This results from the low band dispersion in these compounds (demonstrated by the large effective masses in Table 2), resulting in similar strong excitonic downshifting of the low-energy excitations; *t*_1*u*_(π + σ) → *t*_2*g*_(*d*) and *t*_2*u*_(π) → *t*_2*g*_(*d*) corresponding to ⟨ψ_VBM–1_|*H*′|ψ_CBM_⟩ and ⟨ψ_VBM–2_|*H*′|ψ_CBM_⟩ transitions. Indeed, in their recent paper, Grandhi et al.^37^ refer to the absorption onset of Cs_2_TiBr_6_ as an exciton peak, with our calculations revealing in fact both low-energy peaks to be excitonic in nature. This strong renormalization of the low-energy excitations and the lack of a band-like absorption onset rule out standard spectrum fitting techniques (such as the Tauc and Elliott models) for extracting band gap and exciton binding energies.^55,56^ Notably, the experimental spectra for TiBr_6_^2–^ and TiCl_6_^2–^ salts reported by Brisdon et al.^57^ closely resemble the results for Cs_2_TiBr_6_ and Cs_2_TiCl_6_ reported here and in the literature,^37,38^ evidencing the conclusion of molecular crystal behavior, the orbital assignments of the absorption peaks, and the presence of strong electron–hole interactions. Improved agreement between the calculated and experimental spectra is also found for the Sn compounds upon inclusion of electron–hole interactions, as weaker exciton interactions modify transition intensities and shift spectral weights to give more peak-like absorption onsets. The small residual mismatch in some cases between the *GW*+BSE and experimental spectra could be a result of temperature effects (vibrations can lower the symmetry restriction of dark excitonic transitions in this range), quasiparticle lifetime broadening, or the neglect of higher-order terms in *GW*.^56,58,59^

We highlight that the low-energy absorption peaks for Cs_2_TiX_6_ correspond to charge-transfer Frenkel excitons, with the electron wave function localized on the Ti *t*_2*g*_*d* orbitals (*d*_*xy*_, *d*_*xz*_, *d*_*yz*_) and the hole localized on the surrounding X *p* orbitals of the BX_6_ octahedron. This form of exciton is commonly witnessed in organic and molecular crystals^60^ and has been well-established in other 3*d*^0^ (Ti^4+^, Sc^3+^, and Ca^2+^) halides,^61^ arising here as a consequence of the 0D polyhedral connectivity. The strongly bound nature of these excitons is further demonstrated by the band contributions (“fatband plot”) shown in Figure 6, where the delocalization of the exciton wave functions in reciprocal space for the Ti compounds corresponds to real-space localization of the exciton wavepacket.^41^ The large Stokes shifts (∼0.5 eV) and broad PL emission observed for this family of materials in both this study and other studies^15,21,23^ are other characteristic results of localized bound excitons, as well as strong exciton–phonon coupling and low-energy dark excitons, with the photogenerated electron–hole pair readily localizing within the lattice to yield emissive self-trapped exciton (STE) states.

**Figure 6:**
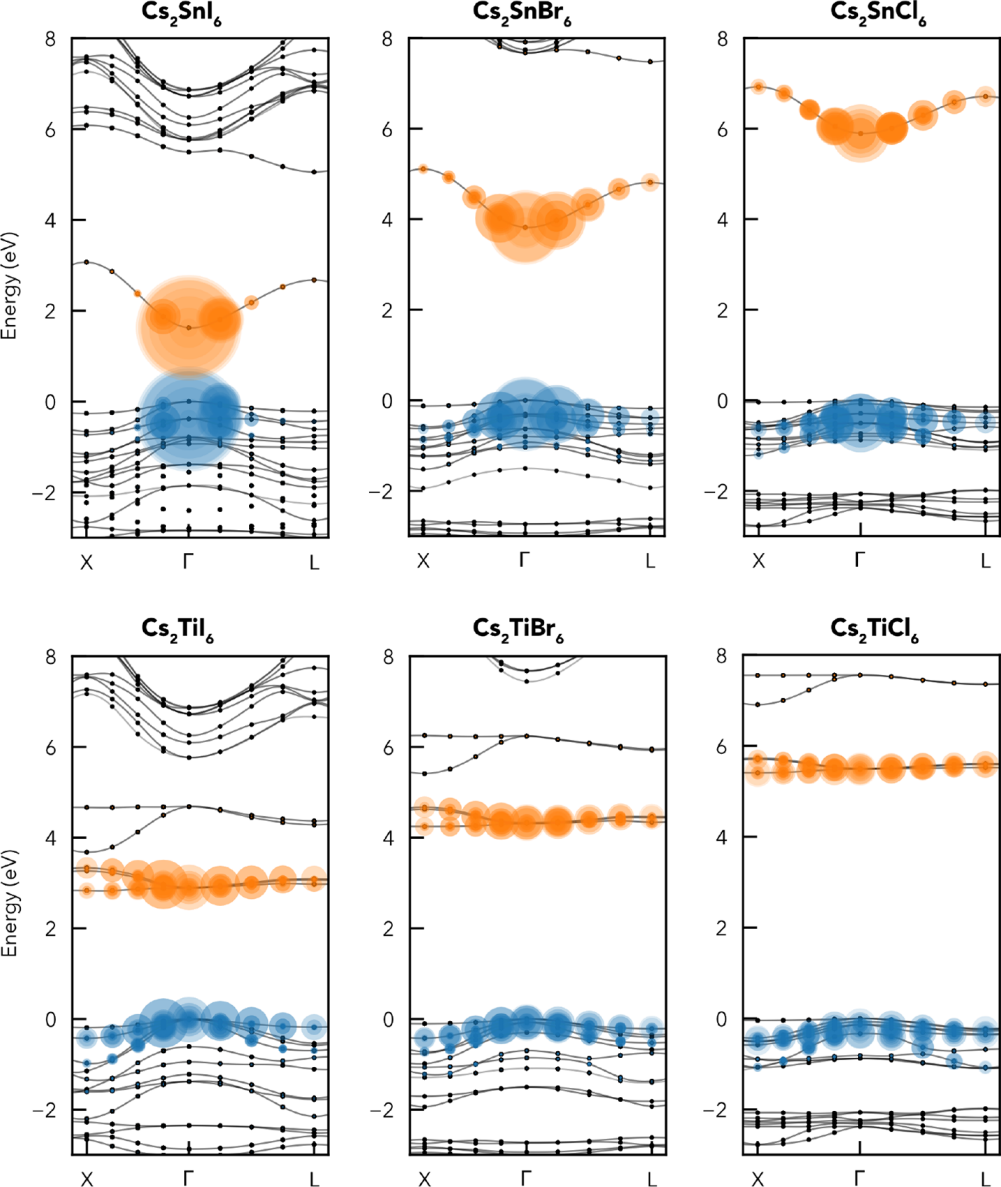
Band contributions to the brightest exciton state at the absorption onset in Cs_2_SnX_6_ (top) and Cs_2_TiX_6_ (bottom), calculated using the BSE approach. Band eigenvalues are indicated by the black dots, with filled circles weighted by their contributions to the exciton state and gray interpolating bands. The average of the three degenerate brightest states at the absorption onset is used, with the sum area of the filled circles normalized across all compositions. Hole and electron states are colored blue and orange, respectively, and the VBM is set to 0 eV.

In contrast, the reduced effective masses of Cs_2_SnX_6_ yield a weakly bound exciton as expected, demonstrated by dominant band contributions at the Γ point to the first bright exciton state. Figure 6 also illustrates the differing trends in band structure when B = Sn and Ti, as X changes from I to Br to Cl, with a greater band gap increase and a reduction in conduction band dispersion for Cs_2_SnX_6_ than Cs_2_TiX_6_ [such that the relative band gap energies of B = Sn and Ti change from X = I (*E*_g, Sn_ < *E*_g, Ti_) to X = Cl (*E*_g, Ti_ < *E*_g, Sn_)], due to the greater localization of the Ti *d* states. As discussed in Section S3, quasiparticle band gaps and thus exciton binding energies from *GW*(+BSE) remain overestimated for these vacancy-ordered compounds, consistent with recent studies that attribute this behavior to underscreening errors within the random phase approximation (RPA) employed within *GW*.^10,62−64^ As such, to avoid this issue and obtain a reasonable estimate of the exciton binding energies in these systems, we also employed a constrained-supercell approach in which an exciton state is generated by controlling spin initialization and band occupation. Here we calculate the exciton binding energy using hybrid DFT for multiple supercell sizes of ≤972 atoms and then extrapolate to the dilute limit using the relevant scaling relationship to avoid supercell-size effects.^65,66^ With this approach, we obtain localized Frenkel exciton states as expected for each Cs_2_TiX_6_ (Figure S13, TOC), with extrapolated binding energies of 0.44, 0.52, and 0.72 eV for X = I, Br, and Cl, respectively (Figure S14), which when subtracted from the HSE06+SOC direct-allowed transition energies in Table 2 brings the hybrid DFT optical transition energy into agreement with the experimental values in each case. For Cs_2_SnX_6_, the electron and hole remain delocalized across the supercell with this approach [under a maximum cell length of 23.1 Å (Figure S13)], yielding extrapolated binding energies close to zero (Figure S15). Further details are provided in Figure S3.3.

Crucially, these results demonstrate the presence of qualitatively different electronic behavior in the Cs_2_SnX_6_ and Cs_2_TiX_6_ families, where despite retaining the same cation valence, the change in frontier-orbital character upon substitution of Sn^4+^ with Ti^4+^ dramatically alters the electronic structure and optical absorption. From the band structures in Figure 6 and values in Table 2, it is clear that the electron effective masses () dictate the exciton behavior in this family, with the weak dispersion and strong real-space localization of the flat d-orbital conduction bands in Cs_2_TiX_6_, aided by the 0D crystal structure, yielding strong electron–hole interactions. This strong excitonic renormalization of the optical absorption in Cs_2_TiX_6_ explains the origin of long-standing discrepancies between experiment and theoretical models of their electronic structure. Moreover, these findings serve as a warning of the changes that can occur when employing ionic substitution as a materials design approach, when such strategies involve changes in valence orbital character.

The presence of strong excitonic interactions in this material family is unsurprising, given the low structural and electronic dimensionality (Figures 1 and 2), weak band dispersion, and large carrier effective masses (Table 2) discussed above. We find the exciton binding strength to be governed by the conduction band character in these compounds, giving the expectation for similar strongly bound Frenkel excitons in A_2_BX_6_ compounds with isoelectronic (*d*^0^) B^4+^ cations, such as Zr and Hf. Indeed, strong excitonic interactions have been recently been reported in Cs_2_ZrX_6_, promising white light emitters,^20^ and a distinct excitonic feature is seen at the absorption onset in Cs_2_HfCl_6_,^67,68^ which has emission and radiation detection applications. Moreover, the bound excitonic behavior in this material class is very similar to that witnessed in the double perovskites,^55,69,70^ which despite a greater structural connectivity, exhibit a low effective electronic dimensionality due to orbital mismatch between the B-site cations.^5,71^ Likewise, extension of theoretical models to include explicit electron–hole interactions was required to reproduce the experimental spectrum,^55,70,72^ explaining the excitonic origin of the direct absorption onset.

In conclusion, by revealing strongly bound excitonic behavior in the cesium titanium halide vacancy-ordered perovskites (Cs_2_TiX_6_), we reconcile long-standing discrepancies between theoretical predictions and experimental measurements for this material class. While previous theoretical studies have found semilocal DFT to yield band gaps matching those from experiment, we show this to be the result of fortuitous error cancellation with qualitatively incorrect absorption spectra and relative band gaps for Cs_2_SnX_6_ versus Cs_2_TiX_6_ (X = I or Br). Our results show that electron–hole interactions are crucial to obtaining the correct polarizability and dielectric screening between octahedra within many-body perturbation theory (MBPT) in these low-electronic-dimensionality systems. A range of optical, photoelectron, and polarization spectroscopies could be employed to further study the behavior of excitons in this material class, including Stark spectroscopy, temperature-dependent optical measurements, and excitation-dependent terahertz and electromodulation spectroscopies.^58,61,69,73^ Moreover, a majority of previous theoretical studies have not included vdW dispersion interactions when modeling these systems, yet here we demonstrate their importance in obtaining accurate crystal and electronic structure predictions, calling for their inclusion in future computational studies of these and related low-dimensional and “molecular” crystals such as the A_4_BX_6_ family.

These findings have important implications for optoelectronic applications. Strong exciton binding can significantly reduce charge separation and open-circuit voltages (*V*_oc_) in solar cells, likely one of the key origins of the poor photovoltaic performance achieved thus far in this material class. Our results show the key role of structural dimensionality and octahedral connectivity, alongside orbital chemistry, in determining the effective electronic dimensionality and optoelectronic properties of inorganic perovskite-inspired materials. More generally, these findings illustrate the importance of considering frontier-orbital character when employing atomic substitution in materials engineering and design strategies, here resulting in qualitatively different electronic behavior despite equal cation valence and similar band gaps.

## Computational Methods

Calculations were performed using both DFT and quasiparticle Green’s function (*GW*) approaches within periodic boundary conditions, through the Vienna Ab Initio Simulation Package (VASP).^74^ Scalar-relativistic pseudopotentials were employed to describe the interaction between core and valence electrons, via the projector-augmented wave (PAW) method.^75^ Specifically, the Cs_sv, Sn_d, Ti_pv, I, Br, and Cl VASP PAW potentials were used. The effect of pseudopotential choice and DFT starting point (semilocal vs hybrid) on the *GW* results was tested and found to give qualitatively similar results, with the same large overestimation of band gaps relative to those from experiment (details in Section S3).

Initial guesses for the crystal structures were obtained from the Materials Project, before relaxing the geometry using the HSE06 screened hybrid DFT functional with dispersion corrections.^76^ The plane-wave energy cutoff and Γ-centered *k*-point mesh were sequentially increased using vaspup2.0(77) until the total energies from static calculations were converged to 0.1 meV/atom, giving values of 300 eV and 3 × 3 × 3, respectively (for the nine-atom primitive unit cell, equivalent to a *k*-point density of 0.33 Å^–1^ in reciprocal space). During structural optimization, a convergence criterion of 0.01 eV/Å was imposed on the forces on each atom and the plane-wave energy cutoff was increased to 500 eV, and the final geometries were re-relaxed, to avoid Pulay stress effects. As discussed at the beginning of the results section, the effect of dispersion corrections (Grimmes D3)^78^ on the structural relaxation was tested and shown to be important; therefore, the HSE06+D3 (using the recommended PBE0+BJ parametrizations)^79^ relaxed unit cells were used for all further calculations in this work.

Electronic band structures and independent-particle optical absorption spectra were initially calculated with the HSE06 hybrid DFT functional, including spin–orbit coupling effects (HSE06+SOC) due to the presence of heavy-atom elements (see results and Section S4). For density of states (DOS) and optical calculations, the *k*-point mesh for the primitive unit cell was increased to 8 × 8 × 8 (reciprocal-space density of 0.12 Å^–1^), and the tetrahedron smearing method was used. The number of virtual states in the optical calculations was increased using vaspup2.0(77) until the high-frequency dielectric constant ε_∞_ was converged to a precision of 0.01. Electronic band structure diagrams were generated using sumo.^80^ Carrier effective masses were determined using nonparabolic fitting of the band edges through the effmass(81) package.

Wave functions calculated using HSE06+SOC were used as input orbitals for the *G*_0_*W*_0_(+BSE) calculations. While only having a modest effect on the band gap energies, SOC was found to have a relatively significant effect on the spectral shape above the absorption onset, as shown in Section S4. Convergence with respect to the number of virtual states/empty bands, imaginary frequency and time grid points, and electron–hole excitation pairs was confirmed in each case. Given the lack of symmetry reduction, the requirement for large numbers of virtual states/empty bands (particularly when including spin–orbit coupling effects) and rapid scaling of the computational cost (memory) with *k*-point density in the *G*_0_*W*_0_+BSE calculations, a 3 × 3 × 3 mesh (for the nine-atom primitive unit cell, equivalent to a *k*-point density of 0.33 Å^–1^ in reciprocal space) was the maximum tractable *k*-point mesh for Cs_2_BX_6_ with our computational resources. While converged for the Br and Cl compounds, the greater band dispersion of the iodides (Cs_2_BI_6_) means the spectra are not well converged for this *k*-point density; thus, the “model BSE” approach^82,83^ was employed to reach converged *k*-point meshes of 4 × 4 × 4 and 8 × 8 × 8 for Cs_2_TiI_6_ and Cs_2_SnI_6_, respectively. Further details about the *G*_0_*W*_0_+BSE calculations are given in Section S3.

Details of the experimental synthesis and absorption measurements are provided in Section S1.

**Figure 5:**
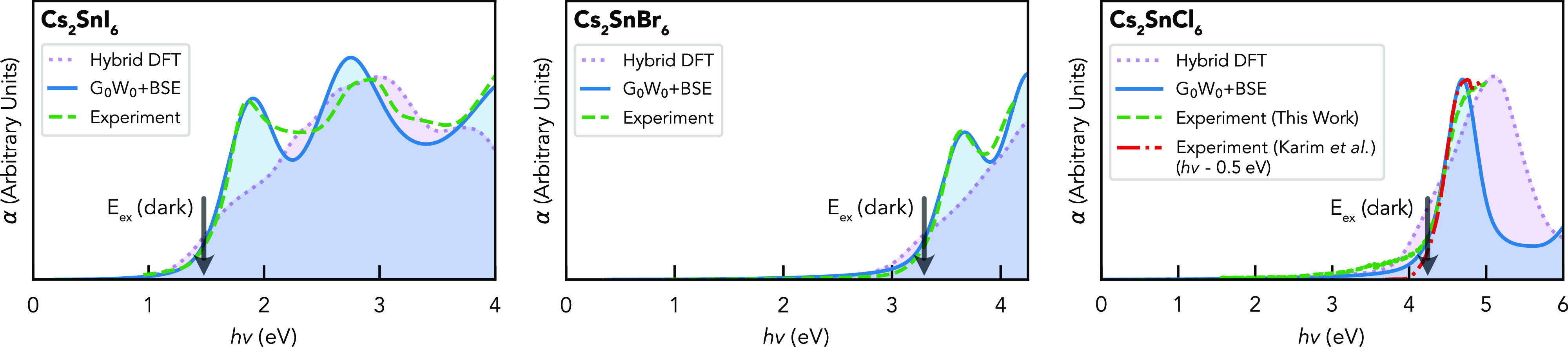
Optical absorption spectra of Cs_2_SnI_6_, Cs_2_SnBr_6_, and Cs_2_SnCl_6_ (from left to right, respectively), calculated with both hybrid DFT (dotted violet) and the *G*_0_*W*_0_+BSE method (solid blue), alongside the experimental data from ultraviolet–visible spectroscopy (dashed green). To directly compare the spectral shapes, calculated curves have been rigidly shifted to match the experimental absorption onset (unshifted results shown in Section S3). For Cs_2_SnCl_6_, the absorption spectrum recorded by Karim et al.^32^ is also shown for comparison (downshifted by 0.5 eV as discussed in Section S3.4).

## Supporting Information Available

Experimental methods, DFT functional dependence of lattice parameters, additional electronic structure analysis, further *GW* calculation details and analysis, and spin–orbit coupling analysis (PDF)

## Notes

Data produced during this work is freely available at doi.org/10.5281/zenodo.7275359.
