# Supplementary material for: Frenkel Excitons in Vacancy-Ordered Titanium Halide Perovskites (Cs2TiX6)
Source: J Phys Chem Lett. 2022 Nov 22;13(47):10965–75. doi: 10.1021/acs.jpclett.2c02436 (PMC9720747; doi:10.1021/acs.jpclett.2c02436)
Supplement: Supplementary file 1 — jz2c02436_si_001.pdf [file jz2c02436_si_001.pdf]

# Frenkel Excitons in Vacancy-Ordered Titanium Halide Perovskites ($Cs_2TiX_6$)

Seán R. Kavanagh,*,†,‡ Christopher N. Savory,† Shanti M. Liga,¶ Gerasimos Konstantatos,¶,§ Aron Walsh,*,‡ and David O. Scanlon*,†

†*Thomas Young Centre and Department of Chemistry, University College London, 20 Gordon Street, London WC1H 0AJ, UK*

‡*Thomas Young Centre and Department of Materials, Imperial College London, Exhibition Road, London SW7 2AZ, UK*

¶*ICFO, Institut de Ciencies Fotoniques, The Barcelona Institute of Science and Technology, 08860 Castelldefels, Barcelona, Spain*

§*ICREA, Institució Catalana de Recerca i Estudis Avançats, 08010 Barcelona, Spain*

E-mail: sean.kavanagh.19@ucl.ac.uk; a.walsh@imperial.ac.uk; d.scanlon@ucl.ac.uk

# S1 Experimental Methods

## S1.1 Chemicals

Cesium acetate (CsAc 99.9% trace metals basis), titanium(IV) isopropoxide (Ti(O-i-Pr)4, ≥97.0%), oleic acid (OlAc, technical grade 90%), bromotrimethylsilane (TMSBr, 97%), chlorotrimethylsilane (TMSCl, ≥99%), 3-(N,Ndimethyloctadecylammonio)propanesulfonate (≥99.0%) and acetone (Z99.5%) were purchased from Sigma-Aldrich. Tin(IV) acetate (SnAc4, 98%) and iodotrimethylsilane (TMSI, 97%) were purchased from Fischer Scientific. Toluene (anhydrous, 99.8%) was purchased from Scharlabs. 1-Octadecene (90%) was purchased from

Alfa Aesar. All chemicals were used without any further purification.

## S1.2 Preparation of cesium oleate solution

Cesium oleate was prepared following a previously reported method.[S1] 0.768 g (4 mmol) of cesium acetate were loaded in a 50 ml three-neck flask together with 7.56 ml of oleic acid and 18.72 ml of octadecene. The solution was degassed through 3 cycles of argon and vacuum and heated at 110 °C for 30 minutes under argon. Afterwards, the solution was left under vacuum for 3 hours at 110°C.

## S1.3 Synthesis of $Cs_2SnX_6$ nanocrystals

For the synthesis of the $Cs_2SnI_6$ NCs, 112.8 mg of Sn(IV) acetate (0.31 mmol) and 60 mg of 3-(N,Ndimethyloctadecylammonio)propanesulfonate (DMOP) were weighed in the glovebox and loaded in a 50 ml three-neck flask together with 4 ml of the cesium oleate solution (0.62 mmol), 2 ml of octadecene and 2 ml of oleic acid. The solution was degassed through 3 cycles of argon and vacuum and heated at 110 °C for 35 minutes under vacuum. At this point, 273 µL of TMSI (1.86 mmol) was injected under argon at 140 °C and the reaction was carried out for 20 seconds and then quenched by diluting the solution with 10 ml of toluene. $Cs_2SnCl_6$ and $Cs_2SnBr_6$ NCs were prepared by following the above procedure except that the Cs:Sn:X precursors ratio used was 2:2:9 instead of 2:1:6, which means that 225.6 mg (0.62 mmol) of Sn acetate were weighted and 360 µL (2.8 mmol) of TMSCl, in the case of $Cs_2SnCl_6$ NCs, and 380 µL (2.8 mmol) of TMSBr, in the case of $Cs_2SnBr_6$, were injected.
To purify the NC solutions, 5 ml of acetone was added to 5 ml of solution already diluted in toluene and centrifuged at 5800 rpm for 10 minutes. The supernatant was discarded and the precipitate redispersed in 3 ml of toluene and centrifuged again at 5800 rpm for 10 minutes. The supernatant was discarded again and the precipitate redispersed in toluene and centrifuged again at 2000 rpm for 4 minutes to remove possible aggregates. Finally, the supernatant was filtered using a 0.22 mm PTFE filter. The solutions were stored under inert

atmosphere.

## S1.4 Synthesis and purification of $Cs_2TiX_6$ nanocrystals

$Cs_2TiBr_6$ and $Cs_2TiI_6$ NCs were prepared following our previously published method according to ref S2 of the manuscript. $Cs_2TiCl_6$ was prepared with the same method, but a higher amount of halide precursor was needed, namely 2.4 ml of TMSCl, while DMOP ligand was not used in the synthesis. The purification of the NC solutions was carried out as for $Cs_2SnX_6$, except that for $Cs_2TiI_6$ and $Cs_2TiCl_6$ NC solutions no antisolvent was added before the first centrifugation.

## S1.5 UV-vis absorption

Optical absorption spectra were collected using a Varian Cary5000 UV-vis-NIR spectrophotometer. When measuring the absorption below 300 nm, the nanocrystals were dispersed in hexane instead of toluene. For the case of $Cs_2SnCl_6$, since the nanocrystals were not stable in hexane, they were deposited on sapphire and the absorption was measured using an internal DRA 2500 integrating sphere.

## S1.6 X-Ray Diffraction

The measured x-ray diffraction patterns for the synthesized $Cs_2BX_6$ (B = Sn, Ti; X = Cl, Br, I) nanocrystals are shown in Figs. S1 and S2. The cubic lattice constants of the $Fm\bar{3}m$ $Cs_2BX_6$ nanocrystals were determined from the measured diffractograms as 11.67 Å, 10.86 Å and 10.40 Å for $Cs_2SnX_6$ (X = I, Br, Cl), and 11.50 Å, 10.72 Å and 10.27 Å for $Cs_2TiX_6$ (X = I, Br, Cl). The fact that these materials are often synthesized in nanocrystalline form (as opposed to thin films or bulk powders) as in this work, likely contributes to the small differences between reported lattice constants in the literature, due to surface/ligand bonding effects.

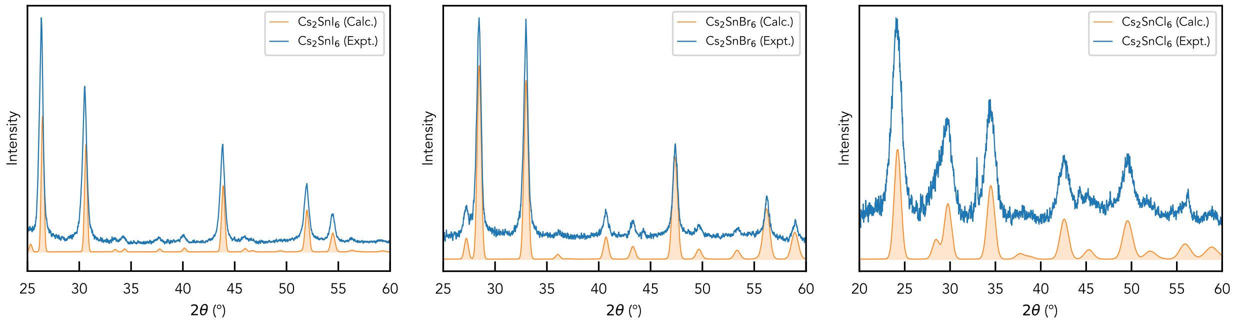


Figure S1: X-ray diffraction patterns for synthesized $Cs_2SnX_6$ (X = I, Br, Cl) nanocrystals. Measured pattern in blue, expected pattern in orange.

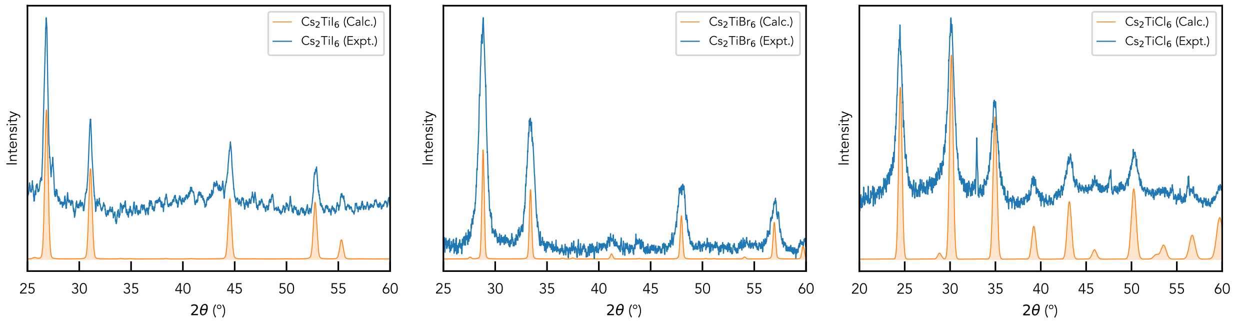


Figure S2: X-ray diffraction patterns for synthesized $Cs_2TiX_6$ (X = I, Br, Cl) nanocrystals. Measured pattern in blue, expected pattern in orange.

Table S1: Calculated cubic lattice parameters for $Cs_2BX_6$ (B = Sn, Ti; X = Cl, Br, I) using semi-local GGA DFT (PBE, PBEsol) and hybrid DFT (HSE06), with and without explicit inclusion of vdW dispersion interactions (D3 correction). Lattice parameter errors ($\Delta a$) given with respect to experimental values.[a]

| | $Cs_2SnCl_6$ | $Cs_2SnBr_6$ | $Cs_2SnI_6$ | $Cs_2TiCl_6$ | $Cs_2TiBr_6$ | $Cs_2TiI_6$ |
|---|---|---|---|---|---|---|
| $a_{PBE}$ | 10.74 Å | 11.26 Å | 12.06 Å | 10.58 Å | 11.08 Å | 11.85 Å |
| $a_{PBEsol}$ | 10.35 Å | 10.82 Å | 11.55 Å | 10.19 Å | 10.63 Å | 11.36 Å |
| $a_{HSE06}$ | 10.65 Å | 11.15 Å | 11.95 Å | 10.51 Å | 10.99 Å | 11.76 Å |
| $a_{PBE+D3}$ | 10.43 Å | 10.90 Å | 11.64 Å | 10.26 Å | 10.71 Å | 11.43 Å |
| $a_{PBEsol+D3}$ | 10.07 Å | 10.51 Å | 11.25 Å | 9.90 Å | 10.31 Å | 11.02 Å |
| $a_{HSE06+D3}$ | 10.32 Å | 10.78 Å | 11.54 Å | 10.18 Å | 10.62 Å | 11.32 Å |
| $a_{Exp}$ | 10.36 Å | 10.77 Å | 11.64 Å | 10.24 Å | 10.68 Å | 11.5 Å |
| $\Delta a_{PBE}$ | 3.7 % | 4.5 % | 3.6 % | 3.3 % | 3.7 % | 3.0 % |
| $\Delta a_{PBEsol}$ | -0.1 % | 0.4 % | -0.8 % | -0.5 % | -0.5 % | -1.2 % |
| $\Delta a_{HSE06}$ | 2.8 % | 3.5 % | 2.7 % | 2.6 % | 2.9 % | 2.3 % |
| $\Delta a_{PBE+D3}$ | 0.7 % | 1.2 % | -0.0 % | 0.2 % | 0.3 % | -0.6 % |
| $\Delta a_{PBEsol+D3}$ | -2.8 % | -2.4 % | -3.4 % | -3.3 % | -3.4 % | -4.2 % |
| $\Delta a_{HSE06+D3}$ | −0.4 % | 0.1 % | −0.9 % | −0.6 % | −0.6 % | −1.5 % |

[a] Experimental values taken from Refs: $Cs_2SnCl_6$,[S3,S4] $Cs_2SnBr_6$,[S4,S5] $Cs_2SnI_6$,[S4,S6–S9] $Cs_2TiCl_6$,[S10,S11] $Cs_2TiBr_6$,[S10–S12] $Cs_2TiI_6$,[S2] matching with our measured values (Section S1.6)

Significant lattice contraction upon inclusion of the D3 dispersion correction is witnessed regardless of the choice of DFT functional (3.2 %, 2.8 % and 3.4 % for PBE, PBEsol and HSE06 respectively). PBEsol, PBE+D3 and HSE06+D3 show the lowest mean absolute errors (MAEs) when compared to experiment, with MAEs of 0.6 %, 0.7 % and 0.5 % respectively. PBE and HSE06 significantly overestimate the lattice parameters (mean signed errors (MSEs) of 3.6 % and 2.8 %) while PBEsol+D3 significantly underestimates the experimental values (MSE of −3.3 %). Given the significant lattice contraction in each case, the low MAE of PBEsol without dispersion corrections is believed likely to be a cancellation of errors.

All computational and experimental data produced during this work is freely available at: `10.5281/zenodo.7275359`.

# S2 Additional Electronic Structure

## S2.1 $Cs_2TiI_6$

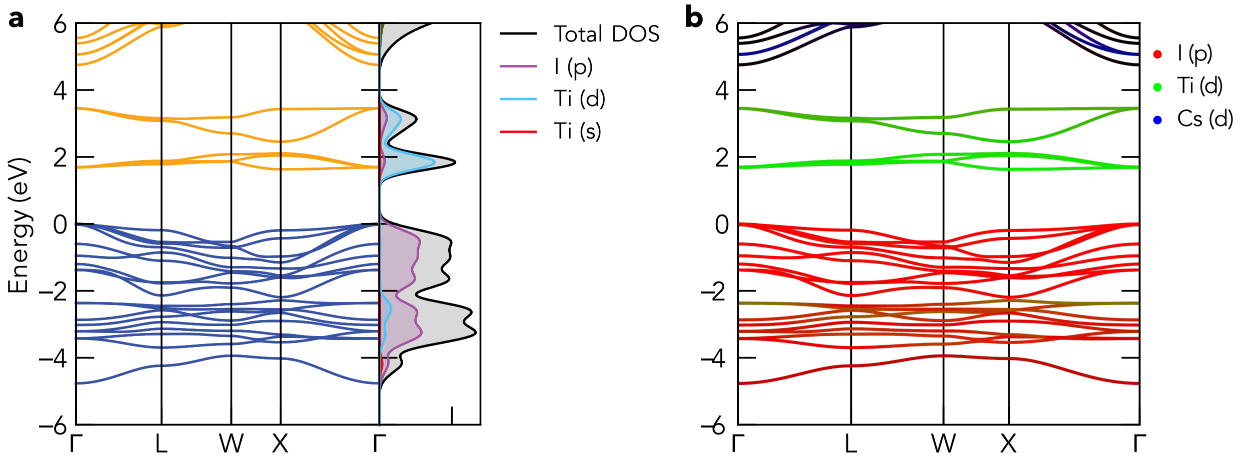


Figure S3: **a.** Electronic band structure of $Cs_2TiI_6$ calculated with hybrid DFT including spin-orbit coupling (HSE06+SOC), alongside a vertical plot of the orbital-projected electronic density of states. Valence band in blue, conduction band in orange, and VBM set to 0 eV. **b.** Orbital-projected electronic band structure of $Cs_2TiI_6$.

## S2.2 $Cs_2TiBr_6$

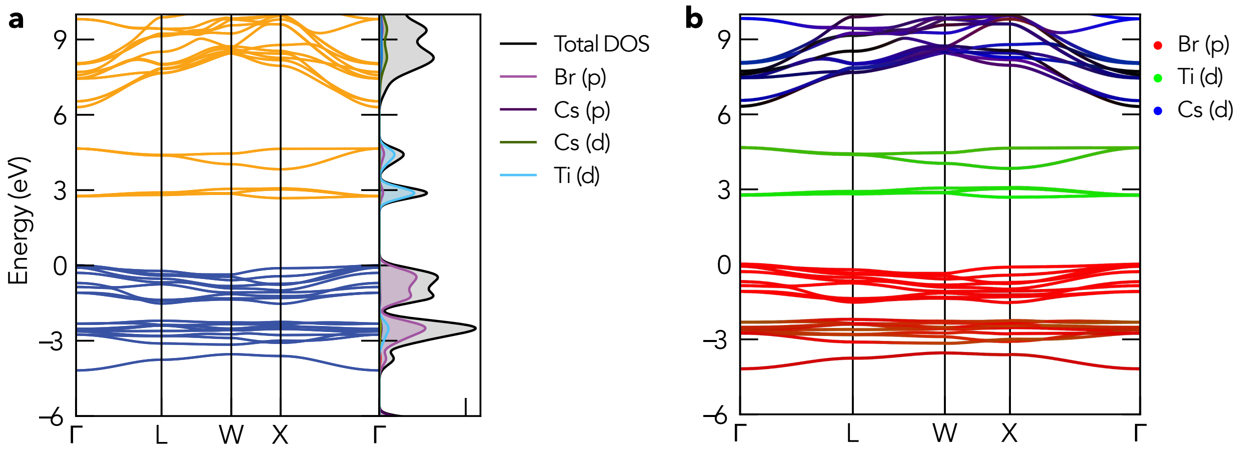


Figure S4: **a.** Electronic band structure of $Cs_2TiBr_6$ calculated with hybrid DFT including spin-orbit coupling (HSE06+SOC), alongside a vertical plot of the orbital-projected electronic density of states. Valence band in blue, conduction band in orange, and VBM set to 0 eV. **b.** Orbital-projected electronic band structure of $Cs_2TiBr_6$.

## S2.3 $Cs_2TiCl_6$

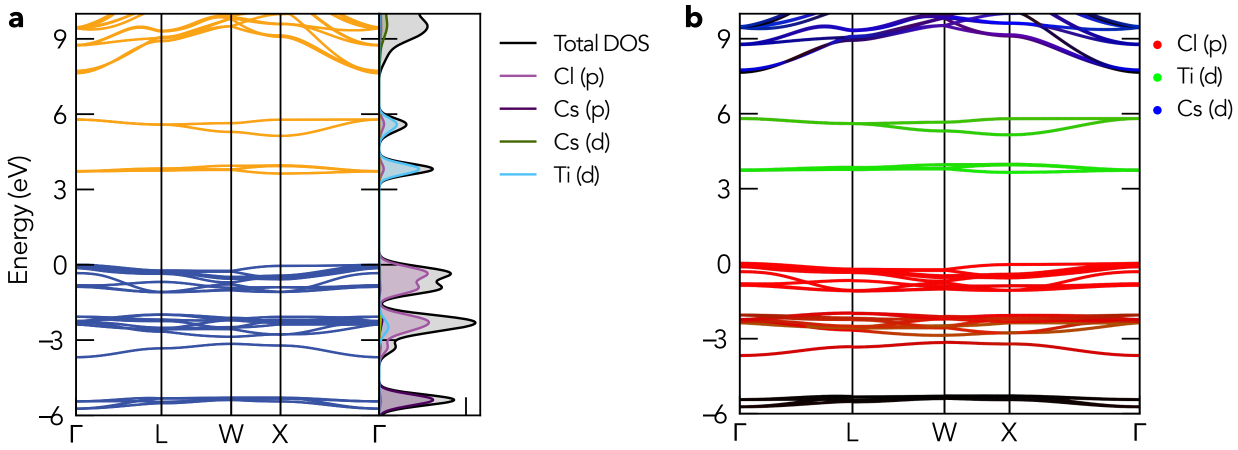


Figure S5: **a.** Electronic band structure of $Cs_2TiCl_6$ calculated with hybrid DFT including spin-orbit coupling (HSE06+SOC), alongside a vertical plot of the orbital-projected electronic density of states. Valence band in blue, conduction band in orange, and VBM set to 0 eV. **b.** Orbital-projected electronic band structure of $Cs_2TiCl_6$.

The deeper electronic levels of the strongly-electronegative Cl anions, and corresponding valence band downshift, results in the appearance of the Cs $p$ states just under 6 eV below the VBM here, as also witnessed for $Cs_2SnCl_6$ (Fig. S8).

## S2.4 $Cs_2SnI_6$

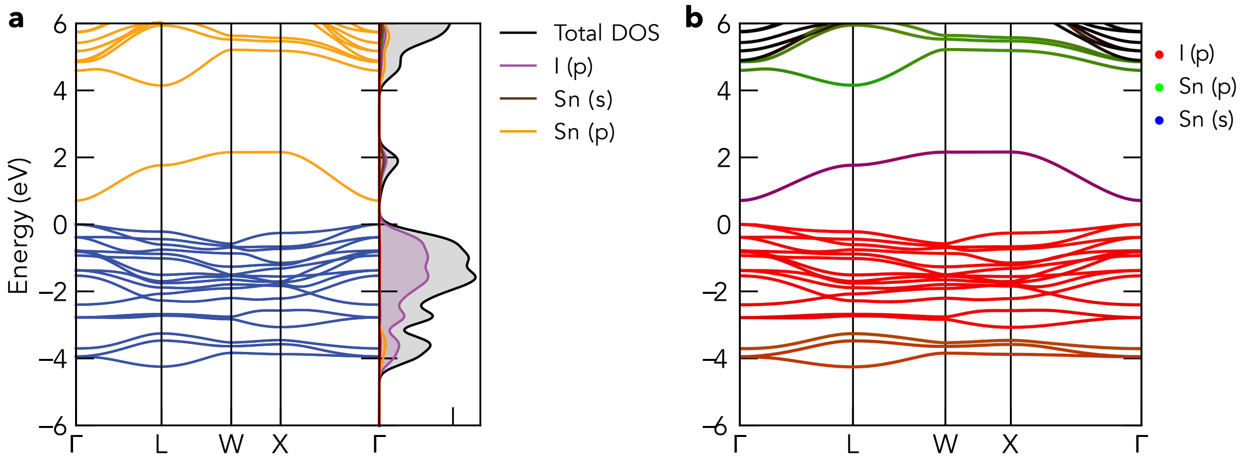


Figure S6: **a.** Electronic band structure of $Cs_2SnI_6$ calculated with hybrid DFT including spin-orbit coupling (HSE06+SOC), alongside a vertical plot of the orbital-projected electronic density of states. Valence band in blue, conduction band in orange, and VBM set to 0 eV. **b.** Orbital-projected electronic band structure of $Cs_2SnI_6$.

## S2.5 $Cs_2SnBr_6$

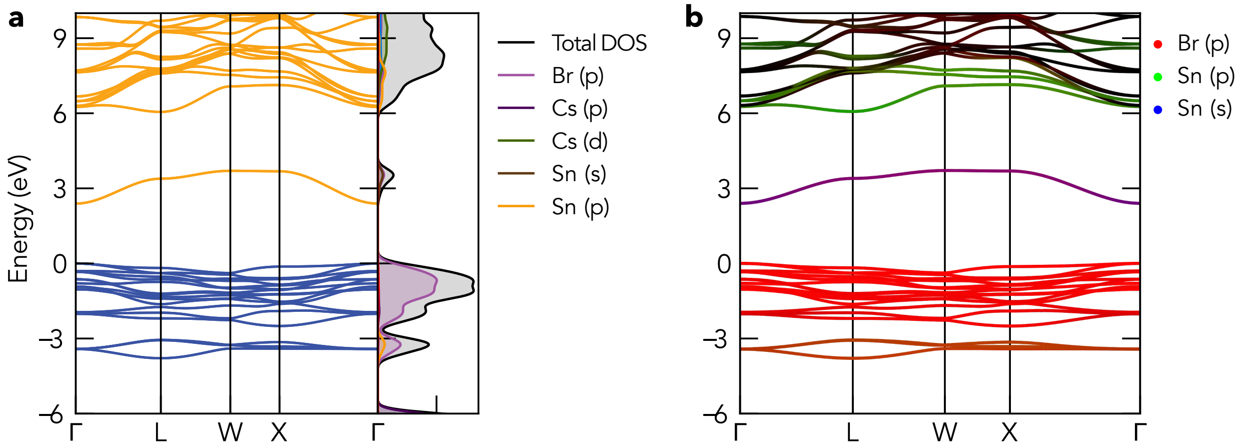


Figure S7: **a.** Electronic band structure of $Cs_2SnBr_6$ calculated with hybrid DFT including spin-orbit coupling (HSE06+SOC), alongside a vertical plot of the orbital-projected electronic density of states. Valence band in blue, conduction band in orange, and VBM set to 0 eV. **b.** Orbital-projected electronic band structure of $Cs_2SnBr_6$.

## S2.6 $Cs_2SnCl_6$

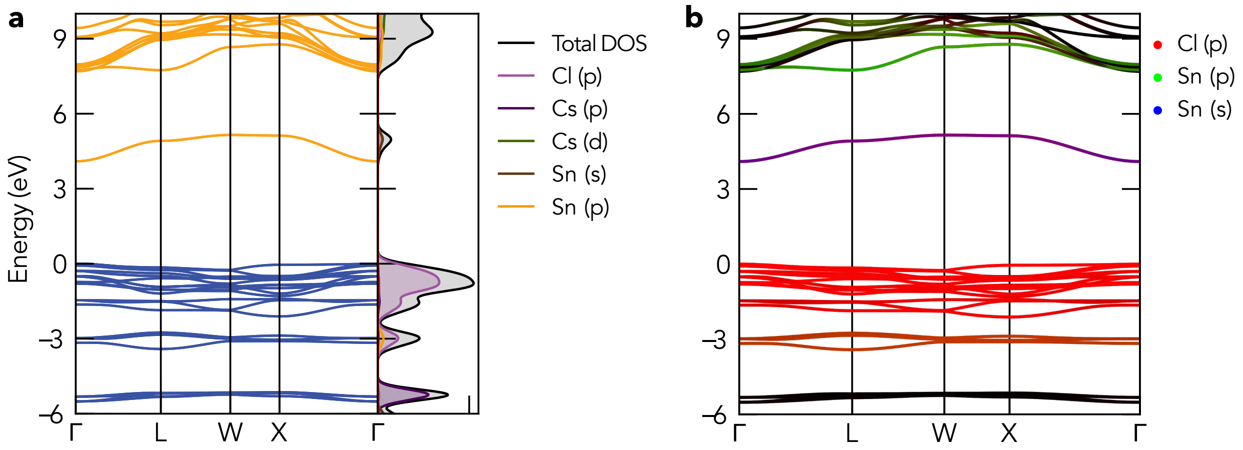


Figure S8: **a.** Electronic band structure of $Cs_2SnCl_6$ calculated with hybrid DFT including spin-orbit coupling (HSE06+SOC), alongside a vertical plot of the orbital-projected electronic density of states. Valence band in blue, conduction band in orange, and VBM set to 0 eV. **b.** Orbital-projected electronic band structure of $Cs_2SnCl_6$.

## S2.7 Additional Notes on the Electronic Structure of $Cs_2BX_6$ (B = Sn, Ti; X = Cl, Br, I)

- The bonding interaction between the metal frontier orbitals with anion $p$ states in the lower valence band ($a_{1g}(\sigma)$ and $e_g(\sigma)$ for Ti $s$ and $d$, $t_{1u}(\sigma)$ for Sn $p$) shows a wider interaction range for Ti than Sn (Figs. S3 to S8), leading to a wider valence bandwidth for $Cs_2TiX_6$ as mentioned in the main text (e.g. 4.8 eV vs 4.3 eV for $Cs_2TiI_6$ and $Cs_2SnI_6$).

- Comparing **????**c, we witness near-identical VBM charge densities as expected, being comprised almost entirely from non-bonding Iodine $p$ states.

- While the shortest anion-anion distances, corresponding to anions within the same $BX_6$ octahedron, show significant differences between $Cs_2SnX_6$ and $Cs_2TiX_6$ (e.g. 4.03 Å vs 3.87 Å for $Cs_2SnI_6$ vs $Cs_2TiI_6$), the second-shortest anion-anion distances, corresponding to anions in neighboring octahedra (i.e. across the vacant octahedral site),

are mostly insensitive to the B cation identity (e.g. 4.13 Å vs 4.14 Å for $Cs_2SnI_6$ vs $Cs_2TiI_6$).

- Much stronger I $p$ contributions to the CBM wavefunctions are witnessed for $Cs_2SnX_6$ (Figs. S6 to S8) than for $Cs_2TiX_6$ (Figs. S3 to S5), reflecting the greater orbital hybridization and thus stronger dispersion in $Cs_2SnX_6$

- Another consequence of mixed ionic-covalent bonding in these systems, similar to other 'perovskite-inspired materials',[S13–S15] is that while the formal oxidation state of B in $A_2BX_6$ is +4, previous work has shown that the strong hybridization of the (formally-unoccupied) B valence orbitals with X $p$ results in an effective ion charge much less than +4.[S16,S17]

# S3 GW Calculations

## S3.1 Further GW Calculation Results

Convergence with respect to the number of empty bands (virtual states) and $k$-point sampling was confirmed in each case, and spin-orbit coupling effects were included in all calculations (which despite a relatively small effect on the band gap are found to significantly affect the absorption shape, particularly for the $G_0W_0$+BSE calculations; Figs. S20 and S21). We note that the quasiparticle band gaps calculated using the $GW$ approach, without explicit electron-hole interactions, are greatly overestimated for all members of the $Cs_2SnX_6$ and $Cs_2TiX_6$ families (Table S3). This behavior was noted by Cucco et al.[S18] for the $Cs_2TiX_6$ family, and attributed to poorly-described correlation effects in the initial DFT Ti $d$ orbitals, however we find this overestimation even with the well-described $Cs_2SnX_6$ family, and in fact worsening when self-consistently iterating the orbitals within the $GW$ calculation (quasiparticle self-consistent $GW$; QS$GW$). This failure of the bare $GW$ approach appears to be a consequence of major under-screening within the Coulomb potential $W$

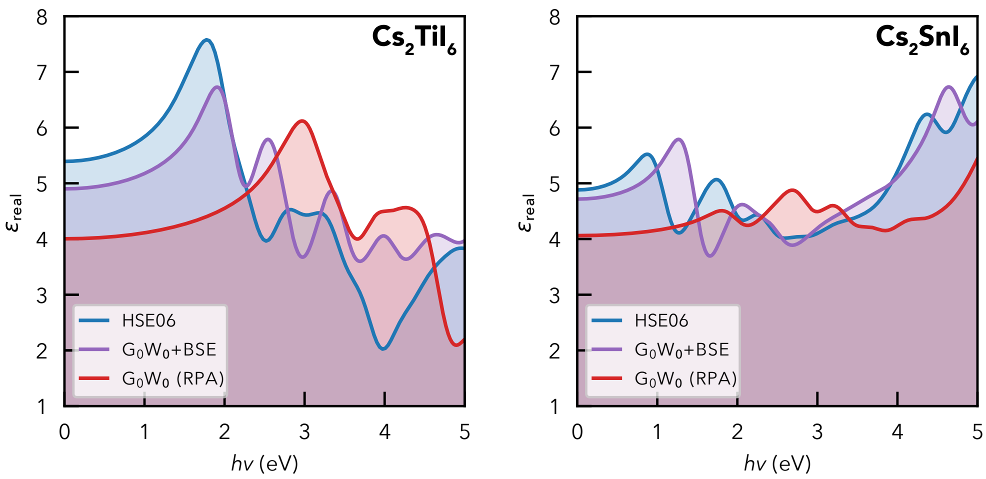


Figure S9: Real component of the dielectric function ($\varepsilon_{real}$) for $Cs_2TiI_6$ (left) and $Cs_2SnI_6$ (right), computed using hybrid DFT (HSE06; blue), $G_0W_0+BSE$ (purple) and the Random Phase Approximation ($G_0W_0$ (RPA); red).

(Fig. S9 and Table S3), which relies on the dielectric function computed via the Random Phase Approximation (RPA).[S19–S22]

This error is known to worsen for localized (e.g. $d/f$-orbital) and lower-dimensional (e.g. vdW-bonded) systems,[S18,S21–S23] thus intensifying here due to the combination of structural, orbital and electronic localization (Fig. 1 and **??**). Perturbative inclusion of ladder diagrams in $W$ (i.e. electron-hole screening and thus excitonic effects) via the BSE, rectifies this overestimation for the Sn-based compounds, with absorption onset energies in good agreement with experiment (Table S3). For the Ti-based compounds however, both the absorption onset and exciton binding energies remain apparently overestimated (Tables S2 and S3), consistent with recent studies which have shown this under-screening error in $GW$(RPA) to cancel under $GW+BSE$ for weakly-correlated systems, but with incomplete cancellation for similar strongly-correlated $d$-orbital systems, requiring state-of-the-art self-consistent vertex corrections and orbital iteration within the $GW$ calculation to rectify this behavior.[S20–S22]

Table S2: Difference in the lowest energy bright excitation calculated using $G_0W_0$ and $G_0W_0 + BSE^a$, to estimate the exciton binding energy

| | $Cs_2SnCl_6$ | $Cs_2SnBr_6$ | $Cs_2SnI_6$ | $Cs_2TiCl_6$ | $Cs_2TiBr_6$ | $Cs_2TiI_6$ |
|---|---|---|---|---|---|---|
| $\Delta\lvert E_{G_0W_0} - E_{G_0W_0+BSE}\rvert$ | 1.42 eV | 0.79 eV | 0.34 eV | 2.11 eV | 1.60 eV | 0.89 eV |

[a] Hartree (bubble) diagrams are omitted from the solution of the BSE here, while ladder diagrams are kept included, so that only excitonic effects formally contribute to the energy shifts. We note however that bubble diagrams were found to have a minor effect on the $G_0W_0 + BSE$ eigenvalues ($< 0.1$ eV).

From Table S2, the estimated exciton binding energies from $G_0W_0$+BSE agree with the qualitative trends predicted by the Wannier effective mass model (??); namely that $E_{ex}(Ti) > E_{ex}(Sn)$ for all halides, and $E_{ex}(Cl) > E_{ex}(Br) > E_{ex}(I)$, due to the reduced band dispersion and heavier carrier masses for Sn → Ti and I → Br → Cl. The $G_0W_0$+BSE estimated exciton binding energies are significantly larger than the Wannier model values, due to both the localized Frenkel nature the excitons in $Cs_2TiX_6$ (X = I, Br, Cl) and $Cs_2SnCl_6$ and the aforementioned under-screening errors within $GW$(RPA).

## S3.2 Further GW Calculation Details

As mentioned in the main text, despite a relatively modest effect on the bandgap energies (Figs. S17 to S19), SOC was found to have a significant effect on the spectral shape above the absorption onset, as shown in Figs. S20 and S21. The lack of symmetry reduction, requirement for large numbers of virtual states / empty bands (particularly with the use of relativistic wavefunctions) and rapid scaling of computational memory demand with $k$-point density in the $G_0W_0$+BSE calculations, means that a $3\times3\times3$ mesh (for the 9-atom primitive unit cell, equivalent to a $k$-point density of $0.33\,\text{Å}^{-1}$ in reciprocal space) was the maximum tractable $k$-point mesh for $Cs_2BX_6$ with our computational resources. This reciprocal space sampling density was found to give well-converged absorption spectra for the Br and Cl compounds, however for the iodides ($Cs_2BI_6$; B = Sn, Ti), the greater band dispersion means the spectra are not well converged for this $k$-point density. Thus for the iodides, we employ the 'model BSE' (mBSE) approach as described by Varrassi et al.[S24] and Tal et al.,[S25]

which has proven to be a reliable method for obtaining approximate $GW$+BSE spectra at increased $k$-point densities, with reduced computational cost. This method involves fitting a local model analytic function $\varepsilon^{-1}_{\mathbf{G},\mathbf{G}}(\mathbf{q})$ to the screened Coulomb potential $W$ from the explicit $G_0W_0$ calculations (with the $3 \times 3 \times 3$ $k$-point mesh), of the form:

$$\varepsilon^{-1}_{\mathbf{G},\mathbf{G}}(\mathbf{q}) = 1 - (1 - \varepsilon^{-1}_{\infty})\exp(-\frac{|\mathbf{q} + \mathbf{G}|^2}{4\lambda^2}). \tag{S1}$$

where $\varepsilon_\infty$ is the high-frequency dielectric constant and $\lambda$ is the range-separation parameter. After fitting this model dielectric screening function $\varepsilon^{-1}_{\mathbf{G},\mathbf{G}}(\mathbf{q})$, we then calculate the mBSE absorption spectrum with a $3 \times 3 \times 3$ $k$-mesh to confirm the reproduction of the explicit $G_0W_0$+BSE calculated spectrum with this $k$-mesh. We then recalculate the absorption spectrum with the fitted $\varepsilon^{-1}_{\mathbf{G},\mathbf{G}}(\mathbf{q})$ within the mBSE approach at $8 \times 8 \times 8$ and $4 \times 4 \times 4$ $k$-point meshes for $Cs_2SnI_6$ and $Cs_2TiI_6$ respectively. For $Cs_2TiI_6$, a $4 \times 4 \times 4$ $k$-point mesh is found to give converged results. For $Cs_2SnI_6$, a $8 \times 8 \times 8$ $k$-point mesh is the maximum tractable $k$-point mesh for this approach due to the high memory demand, found to be mostly-converged with respect to $k$-points, though potentially with some small remaining absence of spectral density around ~2.2 eV (**??**). Gaussian broadening of the calculated spectra (using $\sigma = 0.1$ eV for $Cs_2TiX_6$ and $\sigma = 0.15$ eV for $Cs_2SnX_6$ due to the greater dispersion) and a complex shift of $\eta = 0.1$ eV in the Kramers-Krönig transformation of the dielectric function were used in each case.

The $GW$ VASP recommended projector-augmented wave (PAW) pseudopotentials, optimized for excited-state properties, were used for all $GW$ calculations; `Cs_sv_GW`, `Sn_d_GW`, `Ti_sv_GW`, `I_GW`, `Br_GW` and `Cl_GW`; Table S5. In addition, PAW potentials with the largest valence electron configurations available for `VASP` (PAW2; Table S5) were also trialled, using both semi-local (PBE) and hybrid DFT (HSE06) wavefunctions (Table S4). This pseudopotential choice was found to yield the same trends in $G_0W_0$ quasiparticle gaps, $G_0W_0$+BSE excitation energies and binding energies, though with larger absolute values and thus greater

overestimation of bandgap and binding energies in each case. Moreover, the lack of available `Cl_sv_GW` PAW pseudopotential for `VASP` means that these parameters give incorrect relative bandgaps for $Cs_2TiBr_6$ and $Cs_2TiCl_6$, and thus the PAW1 set was used for all other *GW* calculations in this work.

Convergence with respect to the number of virtual states / empty bands, imaginary frequency and time grid points, and electron-hole excitation pairs was confirmed for the *GW* and BSE calculations. Specifically, 11200 single-electron (due to SOC inclusion) bands were used for the *GW* calculations, corresponding to the maximum number of states allowed with the plane-wave basis set corresponding to the converged energy-cutoff of 300 eV, of which 74 and 72 were occupied for B = Sn and Ti respectively. The same converged plane-wave cutoff energy of 300 eV from the DFT calculations was used for the orbital descriptions, with a reduced cutoff of 200 eV for the response functions, and 50 imaginary frequency and time grid points. For explicit BSE calculations, the number of occupied and unoccupied orbitals included in the BSE electron-hole coupling matrix was set to half the number of occupied single-electron bands (36/37 for B = Sn/Ti), and the maximum energy difference of excitation pairs set to 20 eV. For the model BSE calculations with X = I, the minimum number of occupied/unoccupied orbitals (30/10 and 34/26 for B = Sn and Ti) and excitation pair energy difference (10 eV) required to achieve spectral convergence was tested and used in each case. $Cs_2SnI_6$ required significantly less unoccupied orbitals to be included in the BSE calculations due to the greater conduction band dispersion (**????**), 2-electron Sn *s* (rather than 10-electron Ti *d*) lower conduction band (Figs. S3 and S6) and delocalised Wannier-Mott exciton behaviour (**??**). For the $\Delta|E_{G_0W_0} - E_{G_0W_0+BSE}|$ values reported in Table S2, the 'exciton binding energies' are extracted from explicit *GW*+BSE calculations and then linearly extrapolated to $1/N_k \to 0$ ($N_k \to \infty$), though the difference between the $N_k \to \infty$ value and the final explicitly calculated datapoint is $< 0.06$ eV in all cases.

Table S3: Calculated electronic bandgaps ($E_g$)(lowest energy vertical excitations) and high-frequency dielectric constants ($\varepsilon_\infty$) using the $G_0W_0$ and $G_0W_0$+BSE approaches for $Cs_2BX_6$ (B = Sn, Ti; X = Cl, Br, I), with electronic wavefunctions computed with hybrid DFT including spin-orbit coupling (HSE06+SOC). Bandgaps and dielectric constants calculated using hybrid DFT (HSE06+SOC) and reported experimentally are also included for comparison.[1] [2]

| | $Cs_2SnCl_6$ | $Cs_2SnBr_6$ | $Cs_2SnI_6$ | $Cs_2TiCl_6$ | $Cs_2TiBr_6$ | $Cs_2TiI_6$ |
|---|---|---|---|---|---|---|
| $E_{g,\ G_0W_0\text{@HSE06+SOC}}$ | 5.88 eV | 3.82 eV | 1.63 eV | 5.50 eV | 4.29 eV | 2.85 eV |
| $E_{g,\ G_0W_0\text{@HSE06+SOC + BSE}}$ | 4.31 eV | 2.85 eV | 1.09 eV | 3.18 eV | 2.61 eV | 1.91 eV |
| $\Delta\lvert E_{G_0W_0} - E_{G_0W_0+\text{BSE}}\rvert_{\text{@HSE06+SOC}}$ | 1.57 eV | 0.97 eV | 0.54 eV | 2.32 eV | 1.68 eV | 0.94 eV |
| $E_{g,\ \text{Direct, HSE06+SOC}}$ | 4.10 eV | 2.39 eV | 0.71 eV | 3.68 eV | 2.75 eV | 1.69 eV |
| $E_{g,\ \text{Allowed, HSE06+SOC}}$ | 4.38 eV | 2.70 eV | 1.09 eV | 3.79 eV | 2.84 eV | 1.71 eV |
| $E_{g,\ \text{Exp}}$ | 4.4-4.9 eV | 2.7-3.3 eV | 1.25-1.3 eV | 2.8-3.4 eV | 1.8-2.3 eV | 1.0-1.2 eV |
| $\varepsilon_{\infty,\ G_0W_0\text{@HSE06+SOC(RPA)}}$ | 2.47 | 2.90 | 4.06 | 2.64 | 3.07 | 4.00 |
| $\varepsilon_{\infty,\ G_0W_0\text{@HSE06+SOC+BSE}}$ | 2.85 | 3.39 | 4.71 | 3.12 | 3.67 | 4.90 |
| $\varepsilon_{\infty,\ \text{HSE06+SOC}}$ | 2.83 | 3.37 | 4.87 | 3.24 | 3.85 | 5.39 |

The similarity of the values for $\varepsilon_{\infty,\ G_0W_0+\text{BSE}}$ between B = Ti/Sn in Table S3, along with the *lower* hole masses for the contracted Ti compounds (**??**), provides further evidence for the conduction band effective masses being the dominant factor in the exciton binding strength for $A_2BX_6$.

Bandgaps calculated using $G_0W_0$@PBE+SOC (i.e. *GW* calculations using orbitals computed with the PBE+SOC semi-local DFT functional), with PBE+D3 relaxed crystal structures are found to show similar trends to the $G_0W_0$@HSE06+SOC results (Table S4), with slightly reduced $E_{g,\ G_0W_0}$ for $Cs_2SnX_6$ (except for $Cs_2SnI_6$ which is incorrectly predicted to be metallic) and slightly larger $E_{g,\ G_0W_0}$ for $Cs_2TiX_6$ (except for $Cs_2TiCl_6$ where $E_{g,\ G_0W_0}$ is slightly reduced).

Table S4: Comparison of calculated direct electronic bandgaps ($E_g$)(lowest energy vertical excitations) using the $G_0W_0$ and $G_0W_0$+BSE approaches for $Cs_2BX_6$ (B = Sn, Ti; X = Cl, Br, I), using two different PAW pseudopotential valence electron configurations (PAW1 and PAW2, Table S5) and electronic wavefunctions computed with either hybrid (HSE06) or semi-local (PBE) DFT, including spin-orbit coupling (SOC). Bandgaps reported experimentally are also included for comparison.[3]

| | $Cs_2SnCl_6$ | $Cs_2SnBr_6$ | $Cs_2SnI_6$ | $Cs_2TiCl_6$ | $Cs_2TiBr_6$ | $Cs_2TiI_6$ |
|---|---|---|---|---|---|---|
| PAW1@HSE06+SOC | | | | | | |
| $E_{g,\ G_0W_0}$ | 5.88 eV | 3.82 eV | 1.63 eV | 5.50 eV | 4.29 eV | 2.85 eV |
| $E_{g,\ G_0W_0\text{+BSE}}$ | 4.31 eV | 2.85 eV | 1.09 eV | 3.18 eV | 2.61 eV | 1.91 eV |
| $\Delta\lvert E_{G_0W_0} - E_{G_0W_0\text{+BSE}}\rvert$ | 1.57 eV | 0.97 eV | 0.54 eV | 2.32 eV | 1.68 eV | 0.94 eV |
| PAW2@HSE06+SOC | | | | | | |
| $E_{g,\ G_0W_0}$ | 6.25 eV | 4.78 eV | 2.30 eV | 5.50 eV | 5.72 eV | 3.71 eV |
| $E_{g,\ G_0W_0\text{+BSE}}$ | 4.72 eV | 3.67 eV | 1.58 eV | 3.18 eV | 3.81 eV | 2.59 eV |
| $\Delta\lvert E_{G_0W_0} - E_{G_0W_0\text{+BSE}}\rvert$ | 1.53 eV | 1.12 eV | 0.72 eV | 2.29 eV | 1.91 eV | 1.12 eV |
| PAW2@PBE+SOC | | | | | | |
| $E_{g,\ G_0W_0}$ | 5.53 eV | 4.33 eV | Metal | 5.23 eV | 6.08 eV | 4.09 eV |
| $E_{g,\ G_0W_0\text{+BSE}}$ | 4.09 eV | 3.28 eV | Metal | 3.15 eV | 4.28 eV | 3.04 eV |
| $\Delta\lvert E_{G_0W_0} - E_{G_0W_0\text{+BSE}}\rvert$ | 1.44 eV | 1.05 eV | – | 2.08 eV | 1.81 eV | 1.05 eV |
| $E_g$, Exp | 4.4-4.9 eV | 2.7-3.3 eV | 1.25-1.3 eV | 2.8-3.4 eV | 1.8-2.3 eV | 1.0-1.2 eV |

Table S5: Valence electron configurations of the two different `VASP` projector-augmented wave (PAW) pseudopotentials used for $GW$ calculations in this work. Note that no `Cl_sv_GW` PAW pseudopotential is available for `VASP`

| Element | PAW1 | PAW2 |
|---|---|---|
| Cs | `Cs_sv_GW` = $5s^25p^66s^1$ | `Cs_sv_GW` = $5s^25p^66s^1$ |
| Sn | `Sn_d_GW` = $4d^{10}5s^25p^2$ | `Sn_sv_GW` = $4s^24p^64d^{10}5s^25p^2$ |
| Ti | `Ti_sv_GW` = $3s^23p^64s^23d^2$ | `Ti_sv_GW` = $3s^23p^64s^23d^2$ |
| I | `I_GW` = $5s^25p^5$ | `I_sv_GW` = $4s^24p^64d^{10}5s^25p^5$ |
| Br | `Br_GW` = $4s^24p^5$ | `Br_sv_GW` = $3s^23p^63d^{10}4s^24p^5$ |
| Cl | `Cl_GW` = $3s^23p^5$ | `Cl_GW` = $3s^23p^5$ |

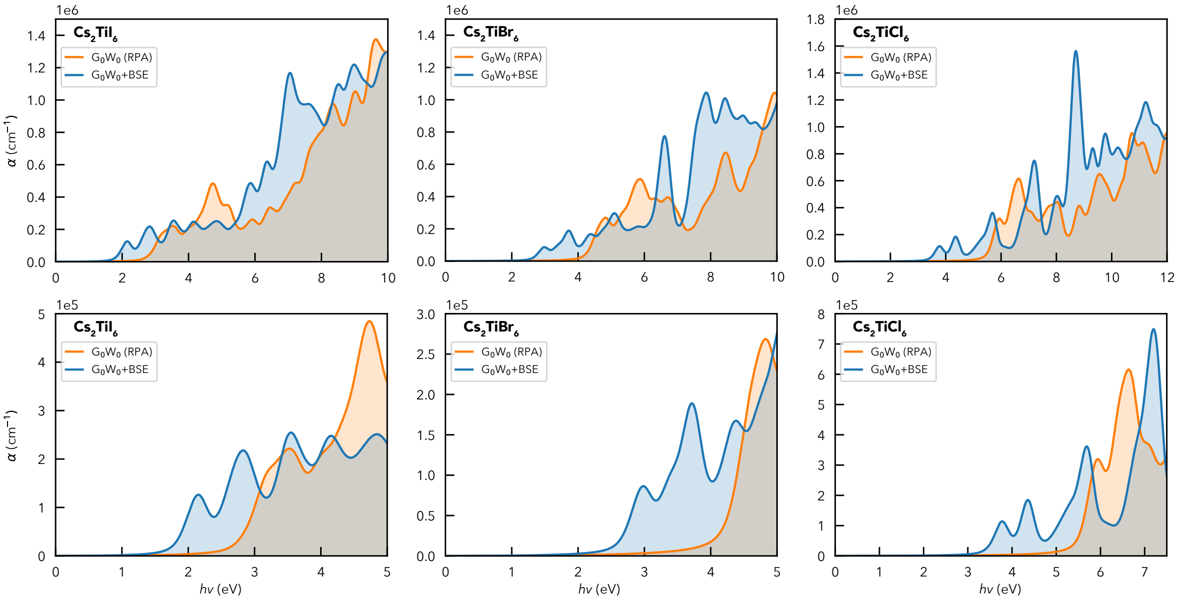


Figure S10: Optical absorption spectra of (left to right) $Cs_2TiI_6$, $Cs_2TiBr_6$ and $Cs_2TiCl_6$, calculated from $G_0W_0$ using the Random Phase Approximation (RPA)(i.e. without electron-hole interactions) in orange and with the Bethe Salpeter Equation (BSE)(i.e. including electron-hole interactions) in blue. The bottom row shows a zoomed-in view of the absorption onset region.

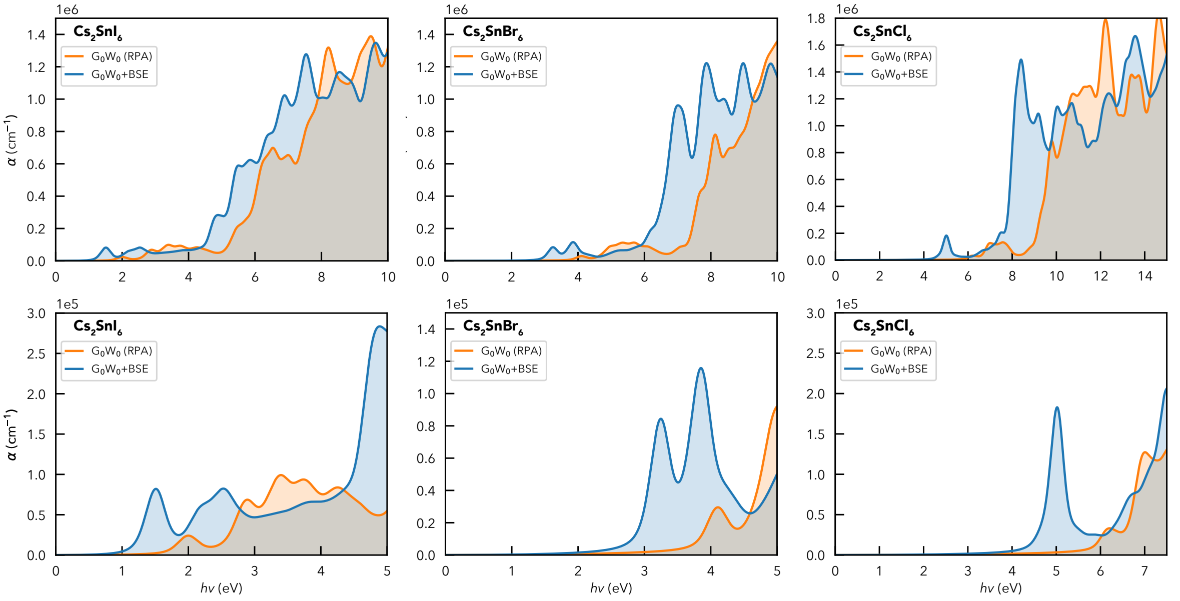


Figure S11: Optical absorption spectra of (left to right) $Cs_2SnI_6$, $Cs_2SnBr_6$ and $Cs_2SnCl_6$, calculated from $G_0W_0$ using the Random Phase Approximation (RPA)(i.e. without electron-hole interactions) in orange and with the Bethe Salpeter Equation (BSE)(i.e. including electron-hole interactions) in blue. The bottom row shows a zoomed-in view of the absorption onset region.

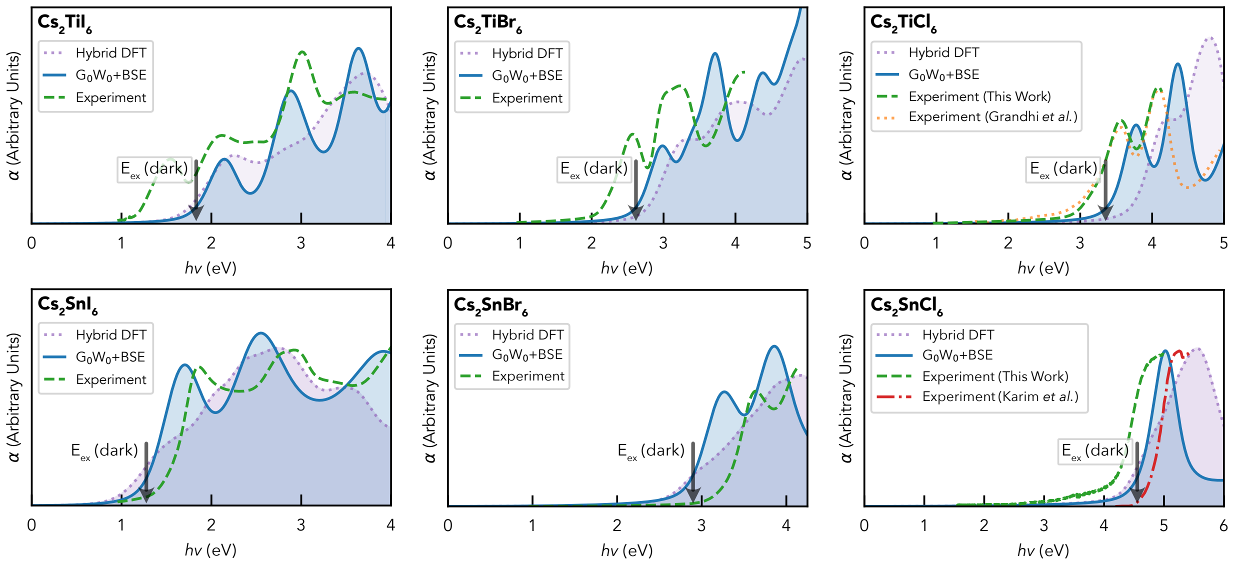


Figure S12: Optical absorption spectra of $Cs_2TiX_6$ **(top)** and $Cs_2SnX_6$ **(bottom)**, calculated with both hybrid DFT (dotted violet) and the $G_0W_0 + BSE$ method (solid blue), alongside the experimental data from UV-Vis spectroscopy (dashed green), with no shifting of the absorption onsets.

## S3.3 Constrained-Supercell Exciton Calculations

Due to the overestimated quasiparticle band gaps and thus exciton binding energies from $GW$(+BSE) for both $Cs_2SnX_6$ and $Cs_2TiX_6$ vacancy-ordered perovskites, a constrained-supercell approach was employed to estimate the exciton binding energies in these systems. Here an exciton state is generated in the supercell by constraining the band occupations to place an electron in a conduction band state and a hole in a valence band state, as well as initialising the atomic spins to have an up-spin magnetic moment on a cation (Sn/Ti) site and down-spin magnetic moments on each of the 6 octahedral-coordinating halides, before relaxing the electronic density using hybrid DFT, while keeping the atom positions fixed. For each X = I, Br, Cl, a localised Frenkel-exciton state is obtained for $Cs_2TiX_6$ (Fig. S13), while fully delocalised states are obtained for $Cs_2SnX_6$ for supercell sizes up to 23.1 Å, as a consequence of delocalised Wannier-Mott exciton behaviour.

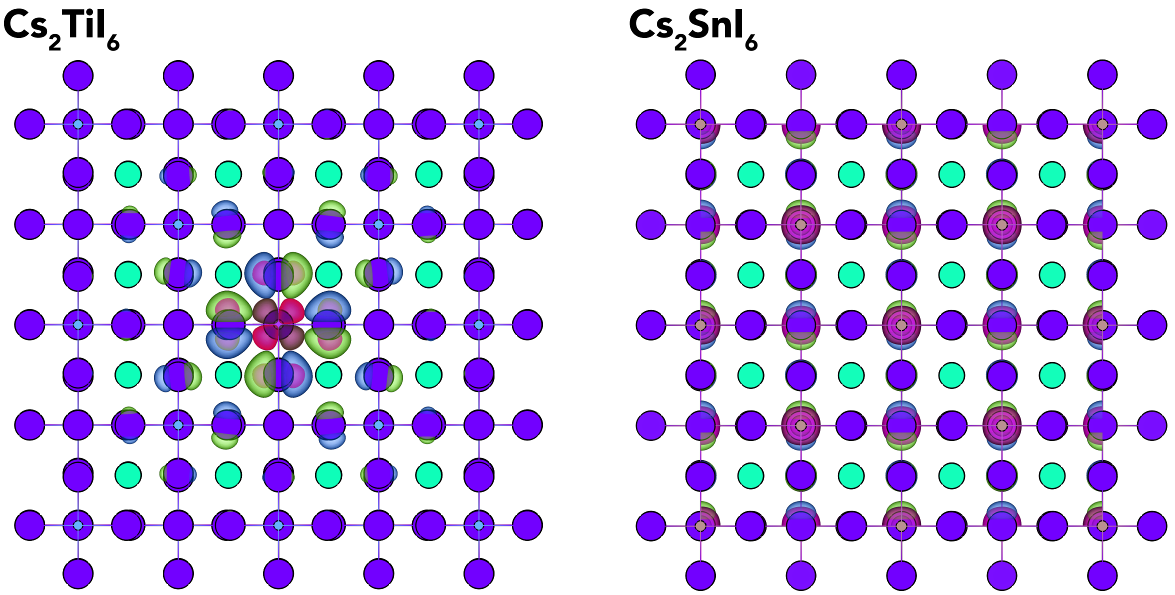


Figure S13: Charge density isosurfaces of the constrained-occupation excitonic supercells for $Cs_2TiI_6$ (left) and $Cs_2SnI_6$ (right). Electron states are shown in dark violet, hole states in light green/blue and an isosurface level of $7 \times 10^{-4}\,\mathrm{e}/\mathrm{\AA}^3$ was used in each case. Caesium atoms in green, titanium/tin in blue/grey, and iodine in purple. Qualitatively similar results were obtained for each X = I, Br, Cl.

The exciton binding energy is then defined as the bandgap minus total energy difference between the constrained-occupation excitonic supercell and the same supercell in the groundstate electronic configuration:

$$E_{\mathrm{ex}} = E_g - (E_{\text{e-h supercell}} - E_{\text{bulk supercell}}) \tag{S2}$$

For $Cs_2TiI_6$, we compute this this exciton binding energy in supercells of 36, 288 and 972 atoms, corresponding to $1\times1\times1$, $2\times2\times2$ and $3\times3\times3$ expansions of the conventional cubic cell, in order to demonstrate the expected linear scaling relationship with respect to inverse cell length of the computed values – a consequence of finite-size point-charge interaction energies.[S35] Extrapolating these linear fits to infinite supercell size in each case, gives predicted exciton binding energies of 0.46 eV, 0.54 eV and 0.74 eV for X = I, Br, Cl in $Cs_2TiX_6$ (Fig. S14). We note that subtracting these values from the HSE06+SOC direct-allowed

transition energies in ?? brings the hybrid DFT optical transition energy into agreement with the experimental values in each case.

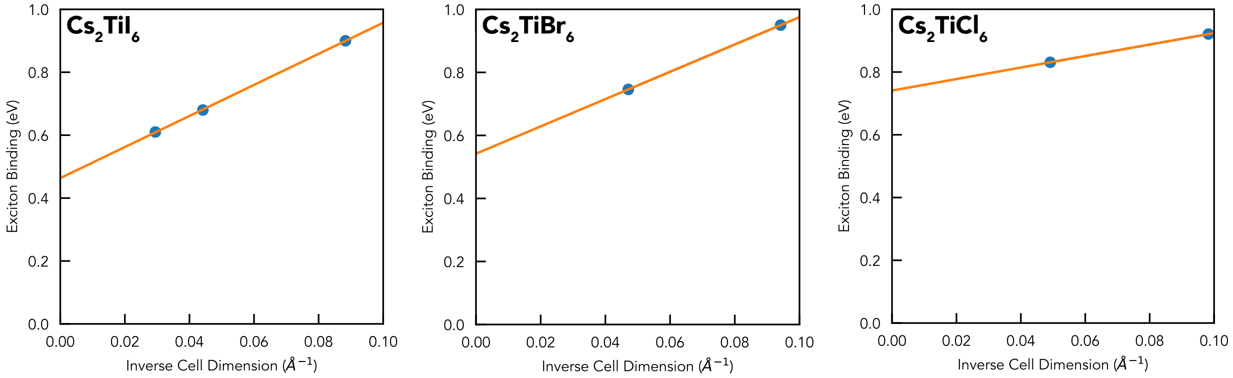


Figure S14: Constrained-occupation supercell exciton binding energies computed using Eq. (S2) with hybrid DFT for $Cs_2TiX_6$. Calculated datapoints in blue, and linear fits as a function of inverse *length* in orange.

For $Cs_2SnX_6$, the electron and hole remain delocalised across the supercell with this approach (under a maximum cell length of 23.1 Å; Fig. S13). The delocalised charge density means the finite-size interactions now scale linearly with inverse *volume*,[S36] indicating delocalised Wannier-Mott exciton behaviour as expected and making this approach unreliable for the calculation of these shallow exciton binding energies. Extrapolating to infinity yields values close to zero (33 meV, 18 meV and 15 meV for X = I, Br, Cl in $Cs_2SnX_6$; Fig. S15).

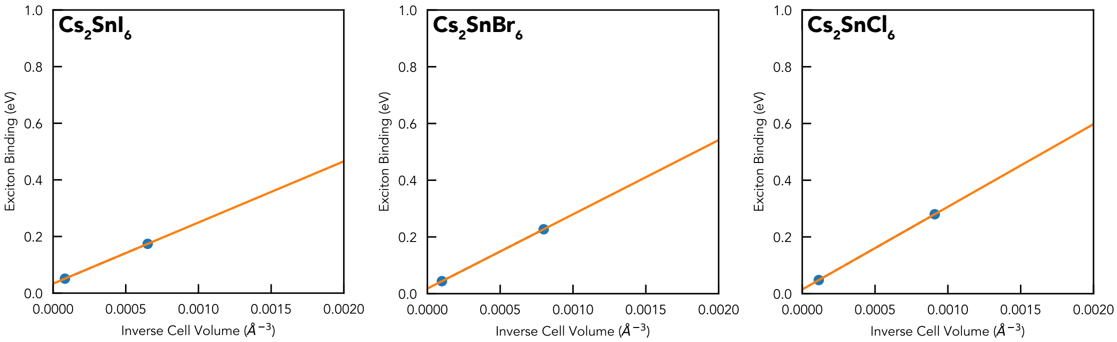


Figure S15: Constrained-occupation supercell exciton binding energies computed using Eq. (S2) with hybrid DFT for $Cs_2SnX_6$. Calculated datapoints in blue, and linear fits as a function of inverse *volume* in orange.

## S3.4 $Cs_2SnCl_6$ Experimental Absorption Spectra

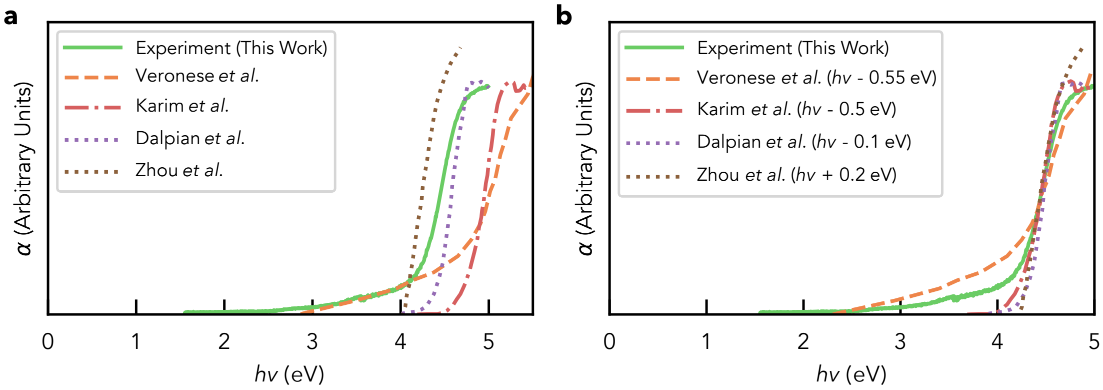


Figure S16: Experimentally-reported[S4,S16,S26,S37] optical absorption spectra of $Cs_2SnCl_6$ **(a)**, and **(b)** rigidly shifted to align the absorption onsets. As most absorption data is reported in arbitrary units, the spectra have been scaled to best match at the onset peak ($E \simeq 4.5\,\text{eV}$).

As noted by Karim et al.,[S4] a relatively wide distribution of reported bandgaps is seen in the literature for $Cs_2SnCl_6$, mostly in the range 4.4 to 4.9 eV (**??**). When digitising and plotting the absorption data reported in previous studies (Fig. S16),[S4,S16,S26,S37] we find that although the measured absorption onset energy varies within a ∼0.7 eV range, the onset shape is relatively consistent between reported samples. Potential origins include differing morphologies of synthesized samples (thin films vs powders vs nanocrystals), quantum confinement and surface/ligand effects in small nanocrystals (and the effect on excitonic binding), as well as the difficulty in measuring optical absorption in the high energy range ($> 4\,\text{eV}$).[S4]

## S4 Spin-Orbit Coupling (SOC) Effects

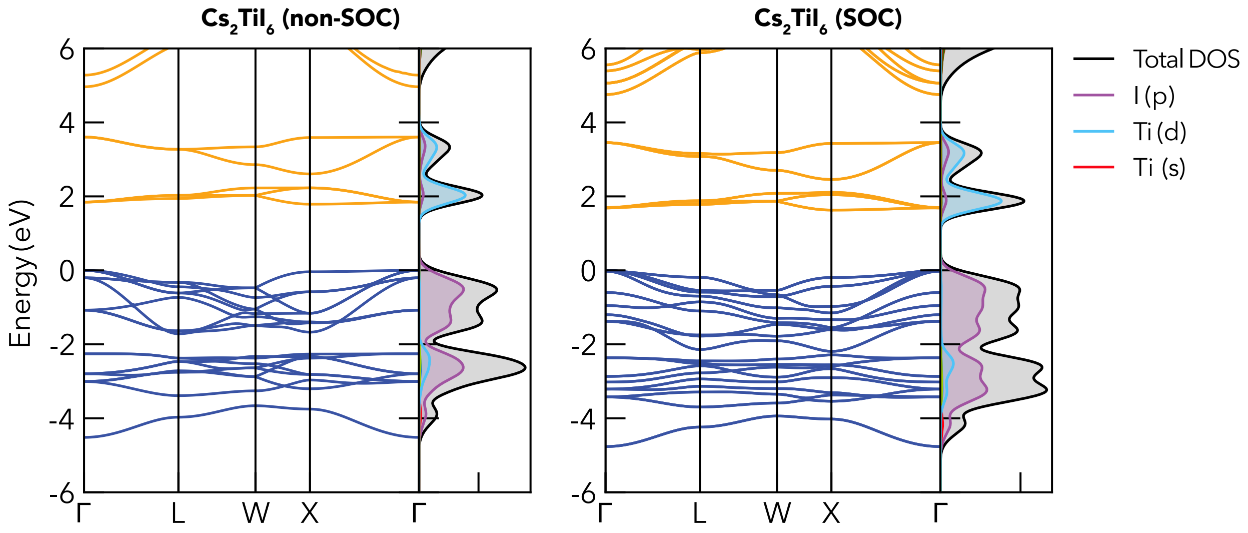


Figure S17: Electronic band structure of $Cs_2TiI_6$ calculated with hybrid DFT (HSE06) excluding ('non-SOC') and including ('SOC') spin-orbit coupling, alongside vertical plots of the orbital-projected electronic density of states. Valence band in blue, conduction band in orange, and VBM set to 0 eV.

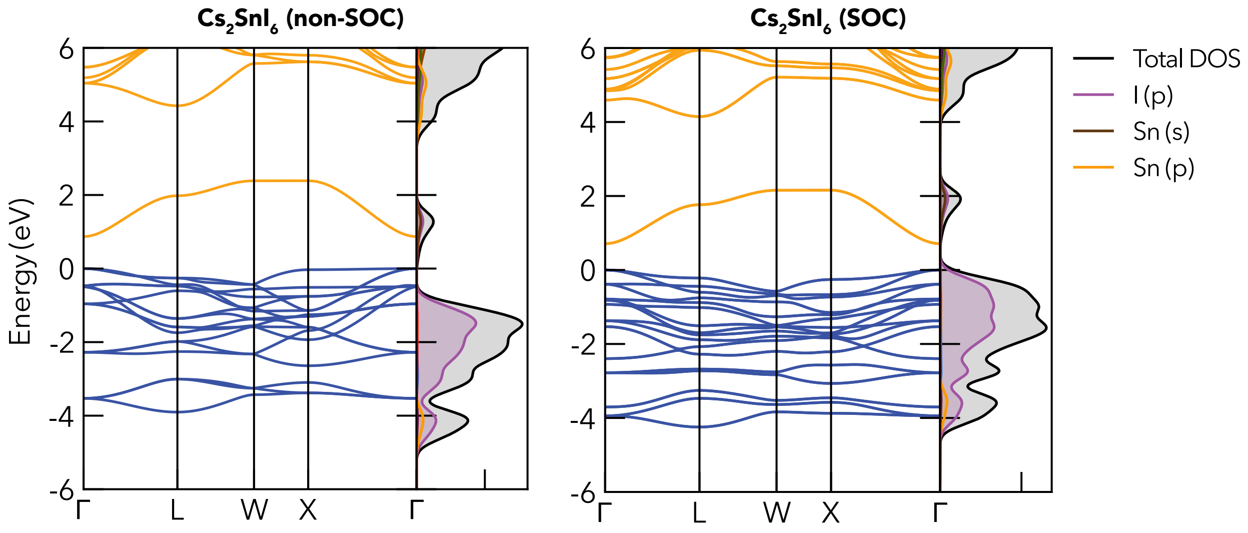


Figure S18: Electronic band structure of $Cs_2SnI_6$ calculated with hybrid DFT (HSE06) excluding ('non-SOC') and including ('SOC') spin-orbit coupling, alongside vertical plots of the orbital-projected electronic density of states. Valence band in blue, conduction band in orange, and VBM set to 0 eV.

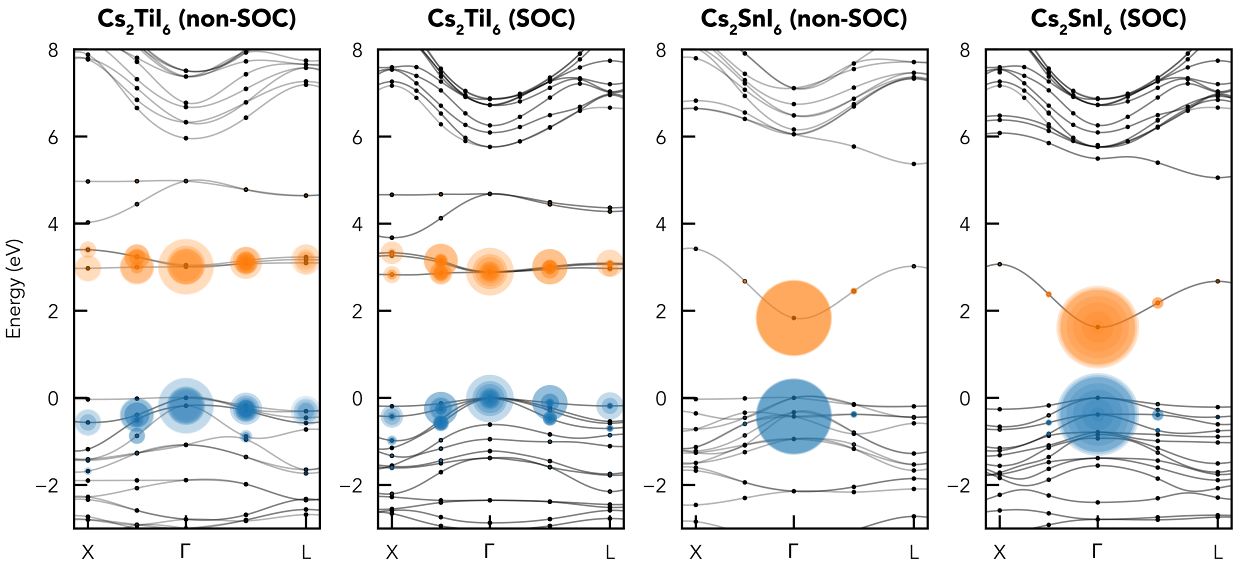


Figure S19: Band contributions to the brightest exciton state at the absorption onset in $Cs_2TiI_6$ **(left)** and $Cs_2SnI_6$ **(right)**, calculated using the $G_0W_0$+BSE approach without ('non-SOC') and with ('SOC') spin-orbit coupling effects. Band eigenvalues are indicated by the black dots, with filled circles weighted by their contributions to the exciton state and gray interpolating bands. The average of the three degenerate brightest states at the absorption onset is used, with the sum area of the filled circles normalized across all compositions. Hole and electron states are shown in blue and orange, respectively, and the VBM is set to 0 eV.

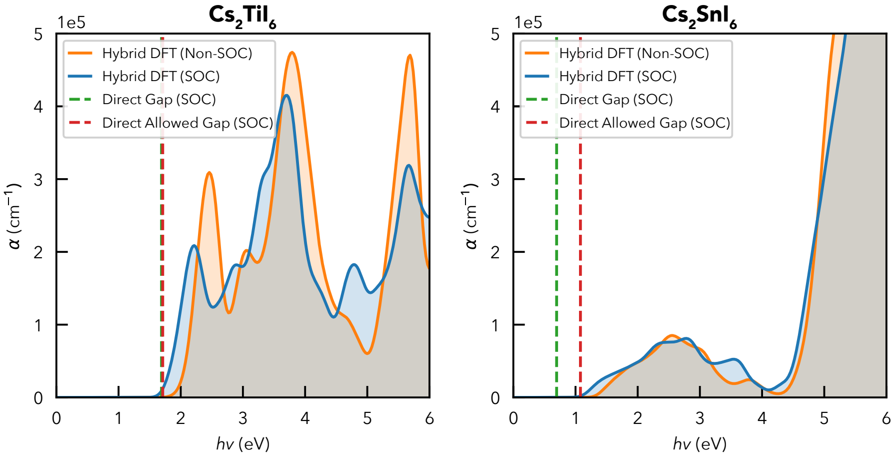


Figure S20: Calculated optical absorption of $Cs_2TiI_6$ (left) and $Cs_2SnI_6$ (right), computed using hybrid DFT (HSE06) excluding ('non-SOC') and including ('SOC') spin-orbit coupling.

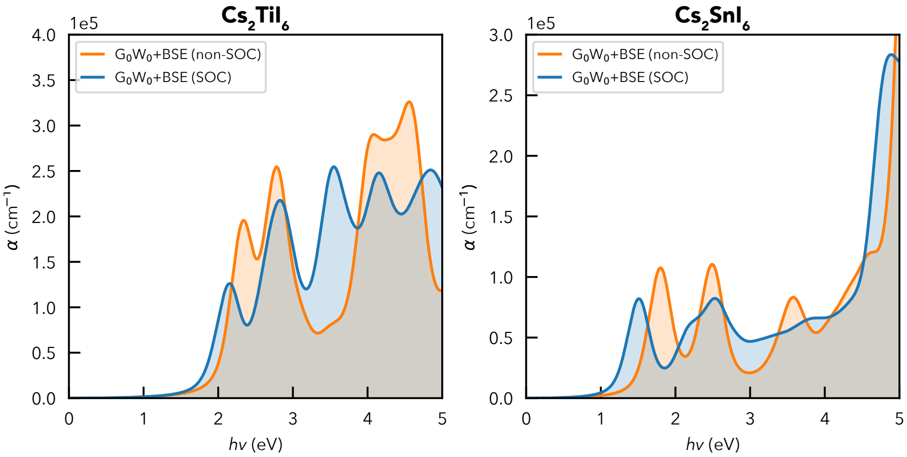


Figure S21: Calculated optical absorption of $Cs_2TiI_6$ (left) and $Cs_2SnI_6$ (right), computed using the $G_0W_0$+BSE approach without ('non-SOC') and with ('SOC') spin-orbit coupling effects.

# References

[S1] Lu, C.; Wright, M. W.; Ma, X.; Li, H.; Itanze, D. S.; Carter, J. A.; Hewitt, C. A.; Donati, G. L.; Carroll, D. L.; Lundin, P. M.; Geyer, S. M. Cesium Oleate Precursor Preparation for Lead Halide Perovskite Nanocrystal Synthe-

sis: The Influence of Excess Oleic Acid on Achieving Solubility, Conversion, and Reproducibility. *Chem. Mater.* **2019**, *31*, 62–67.

[S2] Liga, S. M.; Konstantatos, G. Colloidal Synthesis of Lead-Free $Cs_2TiBr_{6\text{-}x}I_x$ Perovskite Nanocrystals. *J. Mater. Chem. C* **2021**, *9*, 11098–11103.

[S3] Zhu, W.; Xin, G.; Wang, Y.; Min, X.; Yao, T.; Xu, W.; Fang, M.; Shi, S.; Shi, J.; Lian, J. Tunable Optical Properties and Stability of Lead Free All Inorganic Perovskites ($Cs_2SnI_xCl_{6\text{-}x}$). *J. Mater. Chem. A* **2018**, *6*, 2577–2584.

[S4] Karim, M. M. S.; Ganose, A. M.; Pieters, L.; Winnie Leung, W. W.; Wade, J.; Zhang, L.; Scanlon, D. O.; Palgrave, R. G. Anion Distribution, Structural Distortion, and Symmetry-Driven Optical Band Gap Bowing in Mixed Halide $Cs_2SnX_6$ Vacancy Ordered Double Perovskites. *Chem. Mater.* **2019**, *31*, 9430–9444.

[S5] Ketelaar, J. A. A.; Rietdijk, A. A.; van Staveren, C. H. Die Kristallstruktur von Ammonium-, Kalium-, Rubidium- und Cäsiumstannibromid. *Recl. Trav. Chim. Pays-Bas* **1937**, *56*, 907–908.

[S6] Stoumpos, C. C.; Malliakas, C. D.; Kanatzidis, M. G. Semiconducting Tin and Lead Iodide Perovskites with Organic Cations: Phase Transitions, High Mobilities, and Near-Infrared Photoluminescent Properties. *Inorg. Chem.* **2013**, *52*, 9019–9038.

[S7] Lee, B.; Krenselewski, A.; Baik, S. I.; Seidman, D. N.; Chang, R. P. H. Solution Processing of Air-Stable Molecular Semiconducting Iodosalts, $Cs_2SnI_{6\text{-}x}Br_x$, for Potential Solar Cell Applications. *Sustain. Energy Fuels* **2017**, *1*, 710–724.

[S8] Maughan, A. E.; Ganose, A. M.; Candia, A. M.; Granger, J. T.; Scanlon, D. O.; Neilson, J. R. Anharmonicity and Octahedral Tilting in Hybrid Vacancy-Ordered Double Perovskites. *Chem. Mater.* **2018**, *30*, 472–483.

[S9] Kaltzoglou, A.; Antoniadou, M.; Kontos, A. G.; Stoumpos, C. C.; Perganti, D.; Siranidi, E.; Raptis, V.; Trohidou, K.; Psycharis, V.; Kanatzidis, M. G.; Falaras, P. Optical-Vibrational Properties of the Cs $_2$ SnX $_6$ (X = Cl, Br, I) Defect Perovskites and Hole-Transport Efficiency in Dye-Sensitized Solar Cells. *J. Phys. Chem. C* **2016**, *120*, 11777–11785.

[S10] Grandhi, G.; Matuhina, A.; Liu, M.; Annurakshita, S.; Ali-Löytty, H.; Bautista, G.; Vivo, P. Lead-Free Cesium Titanium Bromide Double Perovskite Nanocrystals. *Nanomaterials* **2021**, *11*, 1458.

[S11] Kong, D.; Cheng, D.; Wang, X.; Zhang, K.; Wang, H.; Liu, K.; Li, H.; Sheng, X.; Yin, L. Solution Processed Lead-Free Cesium Titanium Halide Perovskites and Their Structural, Thermal and Optical Characteristics. *J. Mater. Chem. C* **2020**, *8*, 1591–1597.

[S12] Euvrard, J.; Wang, X.; Li, T.; Yan, Y.; Mitzi, D. B. Is $Cs_2TiBr_6$ a Promising Pb-free Perovskite for Solar Energy Applications? *J. Mater. Chem. A* **2020**, *8*, 4049–4054.

[S13] Huang, Y.-T.; Kavanagh, S. R.; Scanlon, D. O.; Walsh, A.; Hoye, R. L. Z. Perovskite-Inspired Materials for Photovoltaics and beyond—from Design to Devices. *Nanotechnology* **2021**, *32*, 132004.

[S14] Kavanagh, S. R.; Savory, C. N.; Scanlon, D. O.; Walsh, A. Hidden Spontaneous Polarisation in the Chalcohalide Photovoltaic Absorber $Sn_2SbS_2I_3$. *Mater. Horiz.* **2021**, *8*, 2709–2716.

[S15] Shi, H.; Du, M.-H. Shallow Halogen Vacancies in Halide Optoelectronic Materials. *Phys. Rev. B* **2014**, *90*, 174103.

[S16] Dalpian, G. M.; Liu, Q.; Stoumpos, C. C.; Douvalis, A. P.; Balasubramanian, M.; Kanatzidis, M. G.; Zunger, A. Changes in Charge Density vs Changes

in Formal Oxidation States: The Case of Sn Halide Perovskites and Their Ordered Vacancy Analogues. *Phys. Rev. Mater.* **2017**, *1*, 025401.

[S17] Ganose, A. Atomic-Scale Insights into Emergent Photovoltaic Absorbers. Doctoral, UCL (University College London), 2018.

[S18] Cucco, B.; Bouder, G.; Pedesseau, L.; Katan, C.; Even, J.; Kepenekian, M.; Volonakis, G. Electronic Structure and Stability of $Cs_2TiX_6$ and $Cs_2ZrX_6$ (X = Br, I) Vacancy Ordered Double Perovskites. *Appl. Phys. Lett.* **2021**, *119*, 181903.

[S19] Shishkin, M.; Marsman, M.; Kresse, G. Accurate Quasiparticle Spectra from Self-Consistent $GW$ Calculations with Vertex Corrections. *Phys. Rev. Lett.* **2007**, *99*, 246403.

[S20] Acharya, S.; Pashov, D.; Rudenko, A. N.; Rösner, M.; van Schilfgaarde, M.; Katsnelson, M. I. Importance of Charge Self-Consistency in First-Principles Description of Strongly Correlated Systems. *npj Comput. Mater.* **2021**, *7*, 208.

[S21] Cunningham, B.; Grüning, M.; Azarhoosh, P.; Pashov, D.; van Schilfgaarde, M. Effect of Ladder Diagrams on Optical Absorption Spectra in a Quasiparticle Self-Consistent GW Framework. *Phys. Rev. Mater.* **2018**, *2*, 034603.

[S22] Cunningham, B.; Gruening, M.; Pashov, D.; van Schilfgaarde, M. QSGW: Quasiparticle Self Consistent GW with Ladder Diagrams in W. *arXiv:2106.05759 [cond-mat]* **2021**,

[S23] Qiu, D. Y.; da Jornada, F. H.; Louie, S. G. Optical Spectrum of $MoS_2$: Many-Body Effects and Diversity of Exciton States. *Phys. Rev. Lett.* **2013**, *111*, 216805.

[S24] Varrassi, L.; Liu, P.; Yavas, Z. E.; Bokdam, M.; Kresse, G.; Franchini, C. Optical and Excitonic Properties of Transition Metal Oxide Perovskites by the Bethe-Salpeter Equation. *Phys. Rev. Mater,* **2021**, *5*, 074601.

[S25] Tal, A.; Liu, P.; Kresse, G.; Pasquarello, A. Accurate Optical Spectra through Time-Dependent Density Functional Theory Based on Screening-Dependent Hybrid Functionals. *Phys. Rev. Res.* **2020**, *2*, 032019.

[S26] Zhou, J.; Luo, J.; Rong, X.; Wei, P.; Molokeev, M. S.; Huang, Y.; Zhao, J.; Liu, Q.; Zhang, X.; Tang, J.; Xia, Z. Lead-Free Perovskite Derivative $Cs_2SnCl_{6-x}Br_x$ Single Crystals for Narrowband Photodetectors. *Adv. Opt. Mater.* **2019**, *7*, 1900139.

[S27] Bonomi, S.; Patrini, M.; Bongiovanni, G.; Malavasi, L. Versatile Vapor Phase Deposition Approach to Cesium Tin Bromide Materials $CsSnBr_3$, $CsSn_2Br_5$ and $Cs_2SnBr_6$. *RSC Adv.* **2020**, *10*, 28478–28482.

[S28] Lee, B.; Stoumpos, C. C.; Zhou, N.; Hao, F.; Malliakas, C.; Yeh, C.-Y.; Marks, T. J.; Kanatzidis, M. G.; Chang, R. P. H. Air-Stable Molecular Semiconducting Iodosalts for Solar Cell Applications: $Cs_2SnI_6$ as a Hole Conductor. *J. Am. Chem. Soc.* **2014**, *136*, 15379–15385.

[S29] Maughan, A. E.; Ganose, A. M.; Bordelon, M. M.; Miller, E. M.; Scanlon, D. O.; Neilson, J. R. Defect Tolerance to Intolerance in the Vacancy-Ordered Double Perovskite Semiconductors $Cs_2SnI_6$ and $Cs_2TeI_6$. *J. Am. Chem. Soc.* **2016**, *138*, 8453–8464.

[S30] Zhang, J.; Yu, C.; Wang, L.; Li, Y.; Ren, Y.; Shum, K. Energy Barrier at the N719-dye/$CsSnI_3$ Interface for Photogenerated Holes in Dye-Sensitized Solar Cells. *Sci. Rep.* **2015**, *4*, 6954.

[S31] Ju, M.-G.; Chen, M.; Zhou, Y.; Garces, H. F.; Dai, J.; Ma, L.; Padture, N. P.; Zeng, X. C. Earth-Abundant Nontoxic Titanium(IV)-Based Vacancy-Ordered Double Perovskite Halides with Tunable 1.0 to 1.8 eV Bandgaps for Photovoltaic Applications. *ACS Energy Lett.* **2018**, *3*, 297–304.

[S32] Chen, M.; Ju, M.-G.; Carl, A. D.; Zong, Y.; Grimm, R. L.; Gu, J.; Zeng, X. C.; Zhou, Y.; Padture, N. P. Cesium Titanium(IV) Bromide Thin Films Based Stable Lead-free Perovskite Solar Cells. *Joule* **2018**, *2*, 558–570.

[S33] He, Y.; Guo, X.; Zheng, H.; Xv, L.; Li, S. Stability Investigation of the Titanium-Based Eco-Friendly Perovskite-like Antifluorite $Cs_2TiBr_6$. *J. Mater. Chem. C* **2022**, *10*, 9301–9309.

[S34] Mendes, J. L.; Gao, W.; Martin, J. L.; Carl, A. D.; Deskins, N. A.; Granados-Focil, S.; Grimm, R. L. Interfacial States, Energetics, and Atmospheric Stability of Large-Grain Antifluorite $Cs_2TiBr_6$. *J. Phys. Chem. C* **2020**, *124*, 24289–24297.

[S35] Freysoldt, C.; Neugebauer, J.; Van de Walle, C. G. Fully *Ab Initio* Finite-Size Corrections for Charged-Defect Supercell Calculations. *Phys. Rev. Lett.* **2009**, *102*, 016402.

[S36] Swift, M. W.; Peelaers, H.; Mu, S.; Morton, J. J. L.; Van de Walle, C. G. First-Principles Calculations of Hyperfine Interaction, Binding Energy, and Quadrupole Coupling for Shallow Donors in Silicon. *npj Comput Mater* **2020**, *6*, 1–9.

[S37] Veronese, A.; Patrini, M.; Bajoni, D.; Ciarrocchi, C.; Quadrelli, P.; Malavasi, L. Highly Tunable Emission by Halide Engineering in Lead-Free Perovskite-Derivative Nanocrystals: The Cs2SnX6 (X = Cl, Br, Br/I, I) System. *Frontiers in Chemistry* **2020**, *8*.
